# Sexual health and sexual behaviours in Chinese women of varied sexual identities: a sequential mixed methods study

**DOI:** 10.1080/26410397.2026.2624200

**Published:** 2026-02-09

**Authors:** Chanchan Wu, Pui Hing Chau, Jung Jae Lee, Edmond Pui Hang Choi

**Affiliations:** a Research Assistant Professor, School of Nursing, Faculty of Health and Social Sciences, The Hong Kong Polytechnic University, Hong Kong, People’s Republic of China. Postdoctoral Fellow, School of Nursing, Li Ka Shing Faculty of Medicine, The University of Hong Kong, Hong Kong, People’s Republic of China; b Associate Professor, School of Nursing, Li Ka Shing Faculty of Medicine, The University of Hong Kong, Hong Kong, People’s Republic of China; c Associate Professor, Sydney Nursing School, Faculty of Medicine and Health, The University of Sydney, Sydney, Australia; d Associate Professor, School of Nursing, Li Ka Shing Faculty of Medicine, The University of Hong Kong, Hong Kong, People’s Republic of China. *Correspondence*: h0714919@connect.hku.hk

**Keywords:** sexual health, sexual behaviour, Chinese women, sexual and gender minority, mixed methods

## Abstract

Globally, sex research has largely centred on Western contexts and has often overlooked women’s sexuality. In China, there is a dearth of literature on women’s sexual health and behaviours, especially those of sexual and gender minorities. This mixed-methods study addresses this gap by investigating the sexual health, behaviours, and lived experiences of Chinese women with diverse sexual identities. A cross-sectional online survey was conducted in 2021, followed by qualitative interviews in 2022–2023. Overall, 509 women aged 18–56 years participated in the survey, including 250 cisgender heterosexual women, 186 cisgender sexual minority women, and 73 transgender women or individuals assigned female at birth who identify as nonbinary. Additionally, semi-structured interviews were conducted with 33 participants (5 cis-heterosexual women, 18 sexual minority women, 2 transgender women, and 8 nonbinary individuals). Quantitative findings indicated that cis-heterosexual women tended to confirm their sexual identities at an earlier age but initiated sexual practices later than minority participants. Regarding safer sex behaviours, consistent condom use was more prevalent among cis-heterosexual women. In contrast, sexual minority participants, reflecting the diversity of their sexual practices, more often adopted women-controlled safer sex strategies, such as cleaning before sex. Four overarching themes were developed, including (1) women’s sexuality has long been invisible but is changing; (2) diverse sexual identities and complex sexual practices; (3) the bond between sex and self-worth; and (4) disparities in safer sex awareness and practice. These findings underscore the crucial role of sexual identity and cultural context in the articulation and experiences of women’s sexuality and sexual health.

## Plain language summary

The sexual health and sexual behaviours of Chinese women, especially those of sexual and gender minorities, remain insufficiently investigated. These groups represent a notably hidden yet important demographic. This study focused on understanding Chinese women’s sexual health, specific behaviours, and their perceptions and experiences of sexual encounters. We collected both quantitative and qualitative data using an online questionnaire survey and semi-structured voice interviews, respectively. A total of 509 women aged 18–56 years participated in the quantitative analysis, including 250 cisgender heterosexual women and 259 gender or sexual minority participants. Furthermore, 33 participants with varied sexual identities participated in detailed interviews. Our quantitative findings revealed diverse sexual practices and corresponding safer sex strategies among women with different sexual identities. Participants also expressed varied preferences regarding their sexual experiences, such as preferred sexual behaviours and types of condoms. These results were further supported by qualitative interview accounts, which indicated that sexual practices often occurred alongside the exploration of sexual identities. Our qualitative analysis developed four main themes. One was the long-standing invisibility of Chinese women’s sexuality that is now changing. Others emerged from the subtle relationship between diverse sexual identities and complex sexual behaviours. These findings provide valuable insights into the experiences and perceptions of sexually and gender-diverse women in China. They highlight the urgent need for tailored, culturally sensitive, and identity-affirming interventions to support their sexual health and well-being.

## Résumé

À l’échelle mondiale, la recherche sur la sexualité s’est largement concentrée sur les contextes occidentaux et a souvent négligé la sexualité féminine. En Chine, les publications sur la santé et les comportements sexuels des femmes, en particulier celles qui appartiennent à des minorités sexuelles et de genre, sont peu nombreuses. Cette étude à méthodes mixtes vise à combler cette lacune en enquêtant sur la santé sexuelle, les comportements et le vécu de Chinoises aux identités sexuelles diverses. Une enquête transversale en ligne a été menée en 2021, suivie d’entretiens qualitatifs en 2022-2023. Dans l’ensemble, 509 femmes âgées de 18 à 56 ans ont participé à l’enquête, dont 250 femmes cisgenres hétérosexuelles, 186 femmes cisgenres appartenant à des minorités sexuelles et 73 femmes transgenres ou personnes assignées femmes à la naissance s’identifiant comme non-binaires. De plus, des entretiens semi-structurés ont été réalisés avec 33 participantes (5 femmes cisgenres hétérosexuelles, 18 femmes issues de minorités sexuelles, 2 femmes transgenres et 8 personnes non-binaires). Les résultats quantitatifs indiquent que les femmes cisgenres hétérosexuelles ont tendance à affirmer leur identité sexuelle à un âge plus précoce, mais initient leurs rapports sexuels plus tard que les participantes appartenant à des minorités. Concernant les pratiques sexuelles à moindre risque, l’utilisation systématique du préservatif est plus fréquente chez les femmes cisgenres hétérosexuelles. En revanche, les participantes issues de minorités sexuelles, reflétant la diversité de leurs pratiques, adoptent plus souvent des stratégies sexuelles à moindre risque contrôlées par les femmes, comme l’hygiène intime avant les rapports sexuels. Quatre thèmes principaux ont été développés: (1) la sexualité des femmes a longtemps été invisible, mais est en train de changer; (2) la diversité des identités sexuelles et la complexité des pratiques sexuelles; (3) le lien entre sexualité et estime de soi; et (4) les disparités en matière de sensibilisation et de pratique des rapports sexuels à moindre risque. Ces résultats soulignent le rôle crucial de l’identité sexuelle et du contexte culturel dans l’expression et le vécu de la sexualité et de la santé sexuelle des femmes.

## Resumen

Mundialmente, la investigación sexual se ha centrado principalmente en contextos occidentales y a menudo ha hecho caso omiso de la sexualidad de las mujeres. En China, hay una escasez de literatura sobre la salud y los comportamientos sexuales de las mujeres, especialmente los de minorías sexuales y de género. Este estudio de métodos mixtos aborda esta laguna al investigar la salud sexual, los comportamientos y las vivencias de mujeres chinas con diversas identidades sexuales. En 2021, se realizó una encuesta transversal en línea, seguida de entrevistas cualitativas en 2022-2023. En general, 509 mujeres de 18 a 56 años participaron en la encuesta: 250 mujeres cisgénero heterosexuales, 186 mujeres cisgénero de minorías sexuales y 73 mujeres transgénero o personas asignadas el sexo femenino al nacer que se identifican como no binarias. Además, se realizaron entrevistas semiestructuradas con 33 participantes (5 mujeres cisgénero heterosexuales, 18 mujeres de minorías sexuales, 2 mujeres transgénero y 8 personas no binarias). Los hallazgos cuantitativos indicaron que las mujeres cisgénero heterosexuales tendían a confirmar sus identidades sexuales a una edad más temprana, pero iniciaban sus prácticas sexuales más tarde que las participantes minoritarias. En cuanto a los comportamientos de sexo más seguro, el uso sistemático del condón era más prevalente entre las mujeres cisgénero heterosexuales. En cambio, las participantes de minorías sexuales, reflejando la diversidad de sus prácticas sexuales, adoptaban con mayor frecuencia estrategias de sexo más seguro controladas por mujeres, tales como limpiarse antes de tener relaciones sexuales. Se desarrollaron cuatro temas centrales: (1) la sexualidad de las mujeres ha sido invisible desde hace mucho tiempo, pero está cambiando; (2) las diversas identidades y prácticas sexuales complejas; (3) el vínculo entre el sexo y la autoestima; y (4) las disparidades en concienciación y práctica de sexo más seguro. Estos hallazgos subrayan el papel crucial de la identidad sexual y el contexto cultural en la articulación y las experiencias de sexualidad y salud sexual de las mujeres.

## Introduction

According to the World Association for Sexual Health,^1^ sexuality is a central aspect of being human throughout life, encompassing not only sex, gender identity, and sexual orientation, but also pleasure, intimacy, and reproduction. It is a complex and multifaceted construct that can be experienced and expressed in desires, values, behaviours, practices, and relationships.^2^ Sexual health, as defined by the World Health Organization,^3^ is a fundamental aspect of overall health and is characterised as a state of physical, emotional, mental and social well-being in relation to sexuality.^4^ There is international consensus that achieving sexual health requires positive and respectful attitudes towards sexuality and sexual relationships,^5^ as well as healthy sexual function and pleasurable sexual experiences.^6^ Nevertheless, stigma and shame surrounding sexuality and sexual desire still exist worldwide,^2^ posing significant barriers to sexual health.^7^ To achieve and maintain sexual health, it is essential that the sexual rights of all individuals are respected, protected, and fulfilled.^5^ However, research has shown that women are much more likely than men to experience violations of their sexual rights,^8^ highlighting the need for particular attention to women’s sexuality.

Contemporary sex research has predominantly focused on populations that are Western, educated, industrialised, rich, and democratic, often referred to as WEIRD, and has largely centred on male experiences.^9^ This gender-biased sex research, which relies primarily on male experience as a standard for all humans, may result in an incomplete or ambiguous understanding of the sexuality of women and sexual minority individuals.^10,11^ Sexual health remains particularly under-researched in non-Western contexts, especially in Asian countries where cultural norms tend to be less sexually permissive. Such limitations highlight the urgent need to address both gender inequality and the lack of cultural diversity in current sex research.

Globally, heteronormativity continues to dominate mainstream sexual scripts, positioning heterosexual penetrative sex as the normative standard. A meta-synthesis of heterosexual women’s meaning of sexuality found that their views on female sexuality are still largely shaped by patriarchal culture and male-centric norms, placing intercourse at the centre of sexual behaviour.^12^ Consequently, condom use is widely regarded as the primary method for safer sex, a perspective that inadequately addresses the sexual health needs of sexual minority populations. This is particularly obvious for sexual and gender minority women, including cisgender women with non-heterosexual orientations, transgender women, and individuals assigned female at birth who identify as nonbinary. Women who engage in same-sex sexual activity report a wider variety of sexual behaviours,^13^ but infrequent use of protective measures during these activities.^14^ While this may be partly attributable to the lower risk of sexually transmitted infection (STI) among sexual minority women,^15,16^ other influencing factors remain unclear. The lack of comprehensive and specific descriptions of alternative practices in sexual minority populations has hindered efforts to redefine and promote safer sex tailored for these groups.

In China, sexuality remains a sensitive topic. Historically, Chinese sexual culture has been deeply rooted in Confucianism and patriarchal social structures, which have led to the subjugation of women and their relegation to a passive and submissive role in sexual relations.^17^ Within this framework, sexual behaviour was traditionally confined to marriage, with procreation as its primary purpose, and women’s sexuality was strictly regulated and stigmatised outside these boundaries.^18^ The evolution of Chinese women’s sexuality over around three decades, since 1980, can be divided into three stages.^19^ The first stage was characterised by the medicalisation of sex, where men were the primary subjects and women were largely excluded from sexual discourse. The second stage was marked by the emergence of sexology as a social science, during which divorced and unmarried pregnant women were often problematised. In the third stage, research became increasingly theory-driven, and women began to engage with sexuality as a means of empowerment.

Driven by rapid economic development, globalisation, and the rise of digital technologies, China has experienced what many scholars describe as a *sexual revolution.*^17,18,20–22^ This period has been marked by a clear trend towards earlier sex debut, increasing societal tolerance and openness towards diverse sexual activities, and the emergence of feminist discourses challenging traditional gender norms. Concurrently, women’s role in sexuality has gained greater recognition,^18^ with about two-thirds of women believing that men should not always occupy the dominant position in sex.^23^ Another particularly notable change is the emergence of *Fourth love*,^21,24^ a term from the Chinese context that describes opposite-sex couples in which the woman takes on the traditional male role and the man takes on the traditional female role. This group is seen as a sexual minority within the broader category of heterosexuality, as it actively challenges conventional heterosexual relationship paradigms and emphasises the dominance of women’s sexuality and sexual pleasure.

Despite these advances, traditional Confucian values and the enduring patriarchal system continue to exert a strong influence. Many Chinese people, especially women, still hold or are subject to conservative attitudes regarding sexuality.^18,25^ This adherence to sexually conservative values can lead to women’s sexual dissatisfaction,^26^ which is associated with lower psychological well-being.^27^ As a result, compared to men, Chinese women report lower levels of physical and psychological satisfaction with their sex lives,^18^ indicating a persistent gender gap in sexual well-being. With regard to public attitudes towards non-traditional sexual behaviours, Chinese people show the greatest tolerance for premarital sex and the least tolerance for same-sex sexual behaviour.^18^ Addressing these gaps is essential not only for advancing knowledge and promoting sexual well-being among Chinese women, but also for supporting the broader objectives of reducing health inequalities as outlined in the United Nations Sustainable Development Goals, particularly those related to good health and well-being, gender equality, and reduced inequalities.^28^

However, the sexual health of adult women in China has been neglected for decades and remains poorly understood. Existing research has focused almost exclusively on cisgender heterosexual women, often in the context of gynaecological disease.^29^ This lack of attention is even more pronounced for sexual and gender minority women, whose distinct needs are largely overlooked.^30,31^ Even research on Chinese sexual minorities has overwhelmingly centred on males, leaving women under-represented.^31^ While Chinese sexual culture has become more open in certain respects, women belonging to sexual and gender minorities continue to encounter intersecting challenges, including minority stress, stigma, and societal pressure to conform to heterosexual norms.^30,32^ As highlighted in a recent review, sexual and reproductive health remains one of the most significant yet unmet health needs among women with same-sex attraction in China.^30^ Although some studies have identified similarities in sexual desire across women with different sexual orientations,^33^ important gaps persist, particularly regarding the objectives of desire and changes over lifespan.^34^

Despite some progress, little is known about the sexual health and behaviours of sexually diverse women in China, even as women are increasingly recognised as a heterogeneous group rather than a monolithic category defined solely by medical or reproductive roles.^20^ Given that sexual health is a central component of overall well-being, it is essential to understand how Chinese women with diverse sexual identities experience and articulate their sexuality and sexual health. To eliminate gender bias in research and promote inclusive health systems that leave no one behind, greater attention must be given to women and sexual minority populations who have been historically under-represented in research.^28,35^ This study therefore seeks to investigate the sexual behaviour and sexual health of sexually diverse Chinese women, with the aim of contributing to a more nuanced and equitable understanding of sexual health in China.

### The current study

The present study is part of a larger project investigating the holistic health of Chinese women with diverse sexual identities. While previous publications from this project have addressed participants’ mental health, quality of life, social support, and sleep quality,^36,37^ the current study focuses exclusively on sexual health. Given the limited information on sexual health among Chinese women, a mixed-methods approach is essential for this study, as it allows for quantitative analysis while also providing contextual and exploratory individual accounts.

Recognising that no single concept or indicator can fully capture the complexity of female sexuality,^12^ this study draws on the sexual health model^*^^38,39^ to guide its assessment. This model, grounded in a holistic and sex-positive framework, outlines fundamental components of human sexuality, such as sexual identity, sexual function, positive sexuality, safer sex behaviour, and intimate relationships. Accordingly, three validated indicators were selected to quantitatively assess sexual health: female sexual function,^40^ positive sexuality,^41^ and sexual satisfaction,^42^ corresponding to physiological function, sexual attitude, and sexual pleasure, respectively.

Given the private and sensitive nature of sexual topics in China, participants’ survey responses may be influenced by social desirability bias.^43^ To address this, the study also included one-on-one qualitative interviews, which have been shown to reduce such bias.^44^ The semi-structured interview guide was developed based on the sexual health model and the operational framework of sexual well-being^†^,^45^ ensuring coverage of key areas such as sexual identity, intimate relationships, sexual behaviour and pleasure, sexual function and dysfunction, and sexual health. To enhance participant comfort and engagement, the guide was tailored to the Chinese context and refined in response to findings from the quantitative phase.

## Methods

This study applied an explanatory sequential mixed-methods design, a procedure in which two distinct phases are conducted sequentially within a single study.^46^ Specifically, the quantitative cross-sectional online survey was conducted ﬁrst, and the qualitative interviews were conducted as a follow-up. The two phases were connected twice in this study, with the first connection being the revision of the interview guide based on the survey findings, and the second connection being the integration of the mixed data from a more holistic perspective. The STROBE^47^ and SRQR^48^ checklists were used for reporting quantitative and qualitative study findings, respectively. In addition to employing both quantitative and qualitative methods, this study adopted an intersectionality-informed analytical framework.^49–51^ This perspective recognises that sexual health is shaped by multiple, interconnected factors, and that individuals’ life experiences can differ greatly, even among those with similar identities, depending on their unique circumstances. Specifically, the study examined personal identity and experiences at the individual level, while also reflecting on broader social positions at the social-structural level,^52^ thereby providing a comprehensive understanding of sexual health among sexually diverse Chinese women.

### Quantitative survey

#### Participants

Eligible participants included Chinese women who were: (1) at least 18 years old; (2) self-identiﬁed as “female”, either cisgender or transgender women; and (3) able to read and understand Mandarin Chinese. Notably, with informed consent as a prerequisite, some individuals assigned female at birth may not identify as female, but their participation was included in this study because they share valuable lived experiences as women. To better illustrate the sexual health of participants with diverse sexuality identities, this study categorised them into three groups: cisgender heterosexual women (cis-heterosexual), transgender women and individuals assigned female at birth who identify as nonbinary (trans/nonbinary), and cisgender sexual orientation minority women (sexual minority).

#### Data collection

Given the sensitivity of sexuality research and the relative invisibility of the sexual minority population, there was no specific sampling frame for this study. In order to approach a diverse sample of participants for the study, both convenience sampling via popular and accessible non-governmental organisations (NGOs) and respondent-driven sampling^53^ were adopted. Specifically, the principal investigator, a cisgender woman, served as a core volunteer in three popular NGOs, including TrueSelf (serving Chinese sexual minorities), Period Pride China (serving Chinese women and any individual experiencing menstruation), and r&B bisexual life (serving Chinese bisexual and pansexual adults). Based on these connections, the research poster for this study could be distributed via all online communities organised by these NGOs.

From July 15 to September 30, 2021, all eligible participants were invited to complete the questionnaire survey via the recruitment poster distributed to different online platforms and communities. In addition, we also posted the recruitment poster on popular social platforms, including WeChat (the most widely used mobile app in China) and Douban (a Chinese platform favoured by sexual minorities and female users). Furthermore, respondent-driven recruitment was used to encourage respondents to help recruit potential peers through their network of connections.^53,54^ Data were collected using an online survey platform (Wenjuanxing), and data collection was initiated after informed consent was obtained. At the end of the survey, participants indicated whether they wanted to be contacted to participate in a follow-up interview by providing their WeChat account or phone number.

#### Measures

Participants who consented to join the study were asked to ﬁll out a set of sociodemographic items, including both the participant’s basic information and their sexuality (gender identity and sexual orientation identity). Specifically, there were three questions about gender identity, namely: “What is your birth-assigned gender?”, “What is the gender on your official documents?”, and “What is your gender identity?” There were also three questions about sexual orientation identity: “What gender attracts you emotionally/romantically?”, “What gender attracts you physically/sexually?”, and “What is your sexual orientation identity?” Given that human behaviours cannot be accurately understood by any single factor, this study also collected information on participants’ other social identities from an intersectional perspective,^55^ such as age, ethnicity, monthly income, educational level, employment status, birthplace, and current residence.

##### Female sexual function index

The female sexual function index (FSFI) is a 19-item self-report questionnaire that measures female sexual function across six domains.^56^ It has been shown to be reliable, with measurement invariance across different sexual orientations.^57^ This study further adapted and validated the Chinese version to make it more inclusive in language and more appropriate in scoring.^29^ The full-scale score is the sum of all six domains, with higher scores indicating better sexual function.

##### New Sexual Satisfaction Scale-Short Form version

The New Sexual Satisfaction Scale-Short Form (NSSS-S) is a 12-item unidimensional scale designed to measure sexual satisfaction and is suitable for sexual minority populations.^58^ With the original author’s permission, this scale was translated into Chinese and culturally validated, and its measurement invariance across sexual identity and age was demonstrated.^59^ The total score is computed by summing all 12 items, with higher scores indicating greater sexual satisfaction.

##### Positive Sexuality Scale

The PSS is a 5-item scale that measures positive sexual expression in adult women.^41^ It has demonstrated good reliability and measurement invariance across the adulthood stage. The total score is derived from the sum of all items, with higher scores indicating greater positive sexuality. This study used the validated Chinese version of the PSS, with permission from both the original author and the Chinese translator.

In addition, we supplemented the survey with 34 items related to sexual behaviour by asking participants about lifetime sexual experience, history of sexually transmitted infections (STIs), related testing behaviours, and safer sex practices. All sexual minority women were asked about their sexuality, identity disclosure status, and their perceptions of marriage. If participants answered that they had sexual experiences with a partner, then they were asked about their sexual activities and behaviours. The complete questionnaire is detailed in the Supplemental file.

#### Data analysis

Quantitative data were analysed using SPSS version 27.0 (IBM Corp). Descriptive statistics (counts and percentages, means and 95% confidence intervals [95% CI]) were used to illustrate participants’ demographic characteristics and sexual health outcomes. There were no missing data in the analysed sample. Recognising that sexual practices can vary widely among different sexual groups, this study did not aim to compare sexual health or behaviours across groups. Instead, the focus was on deepening understanding of the sexual realities of the Chinese majority women, sexual minorities, and trans/nonbinary minorities. Nevertheless, given the importance of age in understanding sexuality, comparisons using one-way ANOVA were conducted among the three groups regarding the mean ages at which participants confirmed their sexual identity and first engaged in masturbation and sexual activity. Additionally, the correlation matrix for the broader set of variables is provided in the Supplemental Table S1.

### Qualitative interviews

#### Participants

The target populations for this qualitative study were Chinese adult women with diverse sexual identities, and the potential interviewees were survey participants who expressed their willingness to participate in follow-up in-depth interviews. Those who indicated interest would be contacted and informed about confidentiality. Only if the interviewees confirmed their consent were interviews conducted. To enrich the exploration of individual experiences and perceptions among sexually diverse women, maximum variation sampling,^60,61^ as a type of purposive sampling method, was used to recruit participants reflecting different sexual identities and sociodemographic backgrounds. Participant recruitment continued until no new findings were identified, reaching a point of informational redundancy with a total of 33 participants.^62–64^

#### Interview guide

The semi-structured interview guide was initially developed based on the sexual health model^38,39^ and the sexual well-being framework.^45^ Given the sensitivity of discussing sex in China,^65^ we anticipated that it might be challenging to motivate participants to talk proactively. To encourage comfort and engagement, we focused on aspects of the model that were more individualised and suited to the Chinese context. Furthermore, the interview guide was updated and refined in response to findings from the quantitative survey. The complete interview guide contained a total of five topics, including sexual health and sexuality; probing questions were added or altered in subsequent interviews based on previous interviews.

The interview guide focused on women’s perceptions and understanding of their own sexual health, using the following open-ended questions: (1) What is your sexual identity and how do you understand it? (2) How do you understand sexual health and how would you describe your sexual function? (3) How do you view sexual pleasure and how do you feel about it? (4) Would you mind sharing your sexual experiences? If you have had any, what was the most memorable one for you? If you haven’t had any, what do you imagine or look forward to experiencing? (5) How do you understand safer sex and how do you ensure your personal sexual safety? (6) What sexual difficulties are you experiencing, and how do you cope with them?

#### Data collection

All interviews were conducted in Mandarin Chinese between May 5, 2022, and March 6, 2023, via phone calls. A specific ID without any personal identifiers was used to ensure participants’ confidentiality throughout the process. Each interview was audio-recorded and transcribed verbatim, ranging from 61 to 283 minutes, with a mean length of 119.7 minutes.

#### Data analysis

Reflexive thematic analysis was used in our qualitative data analysis, as it values the researcher’s subjectivity, can be applied across theoretical frameworks and can be used for both inductive and deductive analyses.^66^ Specifically, this study followed the updated six steps to identify patterns across the data,^67^ and the iterative process was carried out, including 1) data familiarisation and writing familiarisation notes, (2) systematic data coding, (3) generating initial themes from coded and collated data, (4) developing and reviewing themes, (5) refining, defining, and naming themes, and (6) writing up. Throughout these processes, the first author, a female sexual health researcher, was involved in all aspects of data collection and analysis, from developing and piloting the interview guide to conducting each one-on-one interview and listening repeatedly to accurately transcribe all interviews.

Inductive coding was first employed to identify data-driven patterns, followed by selective and deductive coding to organise these patterns into meaningful themes based on the sexual health model and sexual well-being framework. Themes were continually refined through team discussions, which included a sexuality research expert and a bisexual female scholar, drawing on intersectionality and post-modern feminist perspectives to ensure both theoretical coherence and cultural relevance.^51,68^ Ultimately, the process resulted in final themes that captured participants’ lived experiences of either being born a woman or living as a woman, their own perspectives on sexuality, their personal sexual experiences, and their feelings of sexual health. Analysis of the data was conducted in Mandarin using NVivo version 12 (QSR International). The final themes and supporting quotes were translated into English using a rigorous translation and back-translation process, which involved two bilingual researchers proficient in both Mandarin and English.

#### Reflexivity

A researcher occupies multiple positions as both an insider and an outsider, and their shared and differing experiences become a lens for interpretation.^69,70^ The issue of insider/outsider researchers is particularly relevant in sex research.^71^ In this study, all interviews were conducted by the first author, who considers herself mainly an insider as the woman identity with participants (although not all were cisgender) and has been involved in women and sexual minority communities for around five years. Additionally, the first author’s researcher positionality, as an outsider, was acknowledged and reflected upon throughout the study, including in the interpretation of the data. By being aware of the insider/outsider status, the researcher was able to navigate potential biases and better understand the experiences of the participants.

### Mixed data integration

Mixed-methods research involves a deeper and broader methodological orientation.^72,73^ In this study, novel or unanticipated quantitative findings were used to inform and refine the interview guide, enabling more in-depth qualitative exploration and explanation. The results are presented in two stages: first, the quantitative data are reported independently; this is followed by qualitative findings, which illustrate participants’ lived experiences and help clarify or contextualise the quantitative results.

### Ethical considerations

This study was approved by the Human Research Ethics Committee (HREC) of the authors’ university on 8 July 2021, with reference number EA210325. The online questionnaire began after obtaining full informed consent from each participant, and participation was voluntary. Participants could stop or end the questionnaire at any time, but the submission was considered successful only when all required items were completed. Verbal permission to audio-record was obtained again before each one-on-one interview. All information collected during the study was kept anonymous and strictly confidential.

## Results

### Quantitative results

Out of the 524 questionnaires collected, 15 were excluded as they did not meet the eligibility criteria. Specific reasons were that nine respondents were cisgender males, four were under 18 years old, and two provided invalid data due to giving the same answers to conflicting items. Thus, a total of 509 participants (97.1%) were finally included in the quantitative analysis. Regarding their sexual identity, 250 (49.1%) participants were cis-heterosexual women, 186 (36.5%) were sexual minority women, and 73 (14.3%) were transgender women and/or individuals assigned female at birth who self-identify as nonbinary gender.

#### Sociodemographic and sexual experience of the overall participants

Table 1 shows the sociodemographic characteristics and lifetime sexual experience of the overall participants and breakdown by the three groups. The overall sample ranged in age from 18 to 56 years, with a mean age of 25.6 years (95%CI: [25.1, 26.1]). Most participants were in the age group of 21–30 years (68.2%), with only 12 (2.4%) participants being between 41 and 56 years old. The majority had either college or bachelor’s degrees (55.8%) or graduate degrees and above (37.7%), while 6.5% had a high school diploma or less. The majority (91.6%) identified as Han people (ethnic majority), and the remaining 8.4% were ethnic minority individuals. Using gross domestic product (GDP) levels as a reference, most participants (72.3%) were born in provinces with moderate economic development, and 38.1% had moved to areas with higher economic development. Detailed information on participants’ birthplaces and current residence is provided in Supplemental Table S2.

**Table 1.** Sociodemographic and sexual experience of the overall participants (*N* = 509)

Characteristics, *n* (%)	Overall (*N* = 509)	Cis-heterosexual^a^ (*n* = 250)	Sexual minority^b^ (*n* = 186)	Trans/nonbinary^c^ (*n* = 73)
Gender identity				
Cisgender women	436 (85.7)	250 (100.0)	186 (100.0)	/
Transgender women	13 (2.6)	/	/	13 (17.8)
Gender queer	28 (5.5)	/	/	28 (38.4)
Gender fluid	7 (1.4)	/	/	7 (9.6)
Other nonbinary gender^d^	25 (4.9)	/	/	25 (34.2)
Sexual orientation identity				
Heterosexual	259 (50.9)	250 (100.0)	/	9 (12.3)
Homosexual	71 (13.9)	/	51 (27.4)	20 (27.4)
Bisexual	88 (17.3)	/	74 (39.8)	14 (19.2)
Pansexual	52 (10.2)	/	33 (17.7)	19 (26.0)
Other non-heterosexual orientation^e^	39 (7.7)	/	28 (15.1)	11 (15.1)
Mean age [95%CI]^f^				
Mean current age; *p* < 0.001^g^	25.6 [25.1 26.1]	26.8 [26.0 27.5]	24.2 [23.5 24.8]	25.1 [23.7 26.4]
Age of confirmed gender identity; *p* < 0.001^g^	9.1 [8.5 9.7]	6.4 [5.8 7.1]	10.0 [9.0 11.0]	18.3 [16.3 20.3]
Age of confirmed sexual orientation; *p* < 0.001^g^	15.1 [14.6 15.5]	13.2 [12.7 13.8]	17.1 [16.4 17.7]	17.0 [15.3 18.6]
Education level				
High school and below	33 (6.5)	12 (4.8)	13 (7.0)	8 (11.0)
College and Bachelor	284 (55.8)	117 (46.8)	115 (61.8)	52 (71.2)
Graduate degree and above	192 (37.7)	121 (48.4)	58 (31.2)	13 (17.8)
Ethnicity				
Ethnic majority (Han people)	466 (91.6)	224 (89.6)	175 (94.1)	67 (91.8)
Ethnic minority (Hui people, etc.)	43 (8.4)	26 (10.4)	11 (5.9)	6 (8.2)
Employment status				
Unemployed	39 (7.7)	10 (4.0)	17 (9.1)	12 (16.4)
Full-time student	222 (43.6)	104 (41.6)	91 (48.9)	27 (37.0)
Full-time job	195 (38.3)	117 (46.8)	53 (28.5)	25 (34.2)
Others (freelancer)	53 (10.4)	19 (7.6)	25 (13.4)	9 (12.3)
Monthly income, Chinese Yuan (CNY)				
5,000 CNY or less	282 (55.4)	120 (48.0)	119 (64.0)	43 (58.9)
More than 5,000 CNY	227 (44.6)	130 (52.0)	67 (36.0)	30 (41.1)
Birthplace^h^				
Economically developed area	85 (16.7)	35 (14.0)	38 (20.4)	12 (16.4)
Economically moderate area	368 (72.3)	192 (76.8)	125 (67.2)	51 (69.9)
Economically underdeveloped area	56 (11.0)	23 (9.2)	23 (12.4)	10 (13.7)
Current residence^h^				
Economically developed area	186 (36.5)	85 (34.0)	78 (41.9)	23 (31.5)
Economically moderate area	300 (58.9)	153 (61.2)	101 (54.3)	46 (63.0)
Economically underdeveloped area	23 (4.5)	12 (4.8)	7 (3.8)	4 (5.5)
Residence movement^i^				
Upward movers	194 (38.1)	88 (35.2)	79 (42.5)	27 (37.0)
Lateral movers	241 (47.3)	130 (52.0)	76 (40.9)	35 (47.9)
Downward movers	74 (14.5)	32 (12.8)	31 (16.7)	11 (15.1)
Local resident or not				
Local resident	181 (35.6)	85 (34.0)	64 (34.4)	32 (43.8)
Nonlocal resident (Migrant)	328 (64.4)	165 (66.0)	122 (65.6)	41 (56.2)
Relationship				
Not in stable relationship(s)	258 (50.7)	114 (45.6)	104 (55.9)	40 (54.8)
In stable relationship(s)	251 (49.3)	136 (54.4)	82 (44.1)	33 (45.2)
*Relationship types (n* *=* *251)*				
*Monogamous*	*214 (85.3)*	*129 (94.9)*	*62 (75.6)*	*23 (69.7)*
*Non-monogamous*	*37 (14.7)*	*7 (5.1)*	*20 (24.4)*	*10 (30.3)*
Masturbation				
Never	136 (26.7)	100 (40.0)	25 (13.4)	11 (15.1)
Had before	373 (73.3)	150 (60.0)	161 (86.6)	62 (84.9)
*Age of first masturbation, M [95%CI]*^f^; *p* < 0.001^g^	*14.6 [14.0 15.2]*	*16.6 [15.7 17.6]*	*13.2 [12.4 14.0]*	*13.1 [11.8 14.5]*
*Frequency of masturbation (n* *=* *373)*	* *	* *	* *	* *
*Once a week or more*	*143 (38.3)*	*27 (18.0)*	*84 (52.2)*	*32 (51.6)*
*Several times a month*	*118 (31.6)*	*61 (40.7)*	*41 (25.5)*	*16 (25.8)*
*Several times a year*	*56 (15.0)*	*35 (23.3)*	*16 (9.9)*	*5 (8.1)*
*Once a year or less*	*56 (15.0)*	*27 (18.0)*	*20 (12.4)*	*9 (14.5)*
*Sex-toy use (n* *=* *373)*	* *	* *	* *	* *
*Had used before*	*179 (48.0)*	*94 (62.7)*	*57 (35.4)*	*28 (45.2)*
*Never used*	*194 (52.0)*	*56 (37.3)*	*104 (64.6)*	*34 (54.8)*
Had sex with partner(s)				
Never	173 (34.0)	93 (37.2)	57 (30.6)	23 (31.5)
Had before	336 (66.0)	157 (62.8)	129 (69.4)	50 (68.5)
*Age of first sex, M [95%CI]*^f^; *p* < 0.001^g^	*20.4 [20.0 20.7]*	*21.2 [20.7 21.8]*	*19.6 [19.0 20.2]*	*19.5 [18.5 20.6]*
Hookup online				
Never	453 (89.0)	243 (97.2)	152 (81.7)	58 (79.5)
Had before	56 (11.0)	7 (2.8)	34 (18.3)	15 (20.5)

Note: All *p* values were reported by conducting comparisons using Chi-square tests or one-way analysis of variance (ANOVA).

^a^ “Cis-heterosexual” group refers to cisgender heterosexual women (*n* = 250), and the percentages for this column were all calculated with 250 as the denominator.

^b^ “Sexual minority” group refers to cisgender sexual orientation minority women (*n* = 186), and the percentages for this column were all calculated with 186 as the denominator.

^c^ “Trans/nonbinary” group refers to transgender women and/or individuals assigned female at birth while self-identify as nonbinary gender (*n* = 73), and the percentages for this column were all calculated with 73 as the denominator.

^d^ Other nonbinary gender includes gender questioning (someone who is unsure about or is exploring their gender identity), no gender, and other nonbinary genders beyond social norms.

^e^ Other non-heterosexual orientation includes fluid orientation, queer orientation, asexual, grey sexual, gynephilia/femininity, and all other non-heterosexual orientations.

^f^ 95% Confidence interval for mean.

^g^ All *p* values were reported by conducting comparisons using one-way ANOVA.

^h^ According to the gross domestic product (GDP) per capita, “Economically developed areas” refer to provinces where per capita GDP exceeds CNY 100,000. “Economically moderate areas” refer to the provinces where per capita GDP between CNY 50,000 and 100,000. “Economically underdeveloped areas” refer to the provinces where per capita GDP less than CNY 50,000.

^i^ “Upward movers” refer to individuals who moved to more economically developed areas. “Lateral movers” refer to individuals who moved between areas with similar economic levels. “Downward movers” refer to individuals who moved to less economically developed areas.

Regarding the age when their gender identity was confirmed, cis-heterosexual women reported a mean age of 6.4 years (95%CI: [5.8, 7.1]). In contrast, the gender identity occurred at a later age in the two minority groups: 10.0 years [9.0, 11.0] for sexual minority women and 18.3 years [16.3, 20.3] for trans/nonbinary individuals. Likewise, the age of confirmation of their sexual orientation showed a similar pattern, with 13.2 years [12.7, 13.8] for cis-heterosexual women, 17.1 years [16.4, 17.7] for sexual minority women, and 17.0 years [15.3, 18.6] for trans/nonbinary individuals. A total of 373 (73.3%) participants had engaged in masturbatory behaviour, and 336 (66.0%) participants reported having had at least one sexual relationship. Details of specific sexual experiences are presented in Table 1.

#### Sexual characteristics of sexual and gender minority participants

Table 2 presents specific sexual characteristics of both sexual identity minority groups. It is noteworthy that less than half of trans/nonbinary participants’ gender identity process was a natural progression (46.6%), while 19.2% and 13.7% of them experienced feelings of anxiety and depression, respectively. While 72.6% of sexual minority women felt that the exploration of sexual orientation occurred naturally, 8.6–19.4% still experienced various types of psychological distress. In total, only 11.6% had fully disclosed their self-identity, including 10.2% of sexual minority women and 15.1% of trans/nonbinary participants. Table 2 also exhibits information on participants’ perceived sexual role in same-sex relationships, preferred title, and personal views of marriage, with nearly one in five stating that they would never enter into a heterosexual marriage, while a similar proportion stated that they would probably do so.

**Table 2.** Sexual characteristics of gender and sexual minority women (*N* = 259)

Characteristics, *n* (%)	Overall (*N* = 259)	Sexual minority^a^ (*n* = 186)	Trans/nonbinary^b^ (*n* = 73)
Feelings during sexual or gender identity exploration^c^			
Developed naturally and everything went well	169 (65.3)	135 (72.6)	34 (46.6)
Felt anxiety during the process	50 (19.3)	36 (19.4)	14 (19.2)
Felt depression during the process	26 (10.0)	16 (8.6)	10 (13.7)
Felt lonely during the process	41 (15.8)	30 (16.1)	11 (15.1)
Had suicide thoughts but not acted upon	24 (9.3)	16 (8.6)	8 (11.0)
Had suicide thoughts and attempted suicide	7 (2.7)	3 (1.6)	4 (5.5)
Identity disclosure			
Had not come out	46 (17.8)	35 (18.8)	11 (15.1)
Had come out to friends only	79 (30.5)	63 (33.9)	16 (21.9)
Had come out to family members only	12 (4.6)	9 (4.8)	3 (4.1)
Had come out to most people	53 (20.5)	37 (19.9)	16 (21.9)
Had fully come out	30 (11.6)	19 (10.2)	11 (15.1)
Others (Identity in exploration or unsure)	39 (15.1)	23 (12.4)	16 (21.9)
Sexual role in female–female (FF) relationship			
T (Tomboy, masculine-presenting)	14 (5.4)	13 (7.0)	1 (1.4)
P (Pure girl/ Po-“wife”, feminine-presenting)	31 (12.0)	27 (14.5)	4 (5.5)
H (Half, with a more androgynous gender style)	28 (10.8)	27 (14.5)	1 (1.4)
No fixed roles	21 (8.1)	20 (10.8)	1 (1.4)
Prefer not to be labelled	64 (24.7)	64 (34.4)	0
Never involved in FF relationship	101 (39.0)	35 (18.8)	66 (90.4)
Title preference			
拉拉 (Lala, a Chinese loose translation of lesbian)	45 (17.4)	39 (21.0)	6 (8.2)
蕾丝边 (Chinese equivalent of the word lesbian)	22 (8.5)	18 (9.7)	4 (5.5)
同性恋 (Homosexual)	23 (8.9)	16 (8.6)	7 (9.6)
女同志 (Tongzhi, local identity of LGBTQ+)	14 (5.4)	12 (6.5)	2 (2.7)
Women who have sex with women	1 (0.4)	1 (0.5)	0
Bisexual women	52 (20.1)	48 (25.8)	4 (5.5)
Pansexual women	25 (9.7)	20 (10.8)	5 (6.8)
Others (Queer/Straight/Asexual/ …)	27 (10.4)	16 (8.6)	11 (15.1)
Prefer not to be labelled	50 (19.3)	16 (8.6)	34 (46.6)
Personal views on marriage^c^			
Might into a heterosexual marriage	54 (20.8)	40 (21.5)	14 (19.2)
Might marry a gay (fake marriage)	11 (4.2)	5 (2.7)	6 (8.2)
Stand firm to wait the legal same-sex marriage	41 (15.8)	28 (15.1)	13 (17.8)
Will never into a heterosexual marriage	48 (18.5)	34 (18.3)	14 (19.2)
Might choose pre-determined guardianship	76 (29.3)	56 (30.1)	20 (27.4)
No ideas yet	61 (23.6)	46 (24.7)	15 (20.5)
Others (anti-marriage/emigration abroad)	34 (13.1)	27 (14.5)	7 (9.6)

^a^ “Sexual minority” group refers to cisgender sexual orientation minority women (*n* = 186), and the percentages for this column were all calculated with 186 as the denominator.

^b^ “Trans/nonbinary” group refers to transgender women and/or individuals assigned female at birth while self-identify as nonbinary gender (*n* = 73), and the percentages for this column were all calculated with 73 as the denominator.

^c^ Participants could select more than one option.

#### Sexual health and service-related status of the overall participants

Table 3 shows the specific sexual health and service-related status of the overall participants with comparisons. Around one-third (31.2%) of the overall participants experienced one or more abnormal genitourinary symptoms in the last year, including 71 (28.4%) cis-heterosexual women, 63 (33.9%) sexual minority women, and 25 (34.2%) trans/nonbinary individuals. Similarly, about one-third (31.4%) in total had a history of genitourinary disease or STI. Regarding the total scores on the three indicators of sexual health, cis-heterosexual women consistently scored higher. Table 3 also demonstrates participants’ sexual health-related testing, coping options for symptomatic treatment, knowledge of human papillomavirus and corresponding vaccination status, available information and main sources of female sexual health-related knowledge, persons with whom they would like to talk about sex, and the specific sexual knowledge they need, with both similarities and differences among the three groups. Notably, both minority groups expressed greater interest than cis-heterosexual women in female guides to sexual pleasure and guidance on intimate relationships.

**Table 3.** Sexual health and service-related status of the overall participants (*N* = 509)

Characteristics, *n* (%)	Overall (*N* = 509)	Cis-heterosexual^a^ (*n* = 250)	Sexual minority^b^ (*n* = 186)	Trans/nonbinary^c^ (*n* = 73)
Genitourinary abnormal symptoms last year				
No related symptoms	350 (68.8)	179 (71.6)	123 (66.1)	48 (65.8)
Had one or some symptoms	159 (31.2)	71 (28.4)	63 (33.9)	25 (34.2)
History of sexually transmitted infections (STI)				
No related infection history	349 (68.6)	168 (67.2)	129 (69.4)	52 (71.2)
Had infected before	160 (31.4)	82 (32.8)	57 (30.6)	21 (28.8)
New Sexual Satisfaction Scale-Short form (*n* = 336)				
Total score, Mean[95%CI]^d^; *p* = 0.001^e^	40.9 [39.7 42.2]	43.2 [41.5 45.0]	39.8 [37.7 42.0]	36.6 [32.7 40.6]
Positive Sexuality Scale (*n* = 332)				
Total score, Mean [95%CI]^d^; *p* = 0.027^e^	30.1 [29.5 30.8]	31.0 [30.3 31.7]	29.7 [28.4 30.9]	28.6 [26.6 30.6]
Female Sexual Function Index (*n* = 431)				
Total score, Mean [95%CI]^d^; *p* = 0.475^e^	26.9 [26.2 27.5]	27.2 [26.4 28.1]	26.5 [25.2 27.8]	26.3 [24.3 28.2]
History of sexual health-related testing				
Had STI testing before	32 (6.3)	16 (6.4)	13 (7.0)	3 (4.1)
Had gynaecology screening before	198 (38.9)	101 (40.4)	69 (37.1)	28 (38.4)
Had HIV testing before	74 (14.5)	37 (14.8)	24 (12.9)	13 (17.8)
Had Hepatitis B/C testing before	15 (2.9)	10 (4.0)	5 (2.7)	0
Had never tested before	289 (56.8)	142 (56.8)	109 (58.6)	38 (52.1)
Coping options for symptomatic treatment				
Public tertiary hospital	435 (85.5)	218 (87.2)	158 (84.9)	59 (80.8)
Community or private hospital	7 (1.4)	5 (2.0)	5 (2.7)	4 (5.5)
Others (Drug store/do nothing)	15 (2.9)	27 (10.8)	23 (12.4)	10 (13.7)
Sex counselling experience				
Had before	22 (4.3)	9 (3.6)	10 (5.4)	3 (4.1)
No relevant experience	487 (95.7)	241 (96.4)	176 (94.6)	70 (95.9)
Knowledge of human papillomavirus (HPV)				
Know nothing	68 (13.4)	35 (14.0)	18 (9.7)	15 (20.5)
Know some	294 (57.8)	135 (54.0)	120 (64.5)	39 (53.4)
Know most	110 (21.6)	61 (24.4)	38 (20.4)	11 (15.1)
Fully know	37 (7.3)	19 (7.6)	10 (5.4)	8 (11.0)
HPV vaccination status				
Not vaccinated and not intend to do	81 (15.9)	42 (16.8)	27 (14.5)	12 (16.4)
Not vaccinated but will do	252 (49.5)	114 (45.6)	94 (50.5)	44 (60.3)
Vaccinated or in progress	166 (32.6)	90 (36.0)	62 (33.3)	14 (19.2)
Others (Over the vaccination age, etc.)	10 (2.0)	4 (1.6)	3 (1.6)	3 (4.1)
Available female sexual health information				
Never been exposed to such information	113 (22.2)	69 (27.6)	30 (16.1)	14 (19.2)
Have fixed source(s)	139 (27.3)	51 (20.4)	66 (35.5)	22 (30.1)
Have random or other source(s)	145 (28.5)	66 (26.4)	59 (31.7)	20 (27.4)
Not sure where to get such information	112 (22.0)	64 (25.6)	31 (16.7)	17 (23.3)
Main source of sexual information				
Traditional media (Books/TV/Broadcast)	123 (24.2)	67 (26.8)	42 (22.6)	14 (19.2)
New media (Wechat/Weibo/Websites)	270 (53.0)	110 (44.0)	123 (66.1)	37 (50.7)
Friends/Peers/Family/Community	51 (10.0)	31 (12.4)	9 (4.8)	11 (15.1)
Healthcare staff	35 (6.9)	25 (10.0)	5 (2.7)	5 (6.8)
No specific or other sources	30 (5.9)	17 (6.8)	7 (3.8)	6 (8.2)
Sufficiency of existing sexual information				
Cannot meet the needs at all	27 (5.3)	8 (3.2)	12 (6.5)	7 (9.6)
Not quite able to meet needs	154 (30.3)	79 (31.6)	61 (32.8)	14 (19.2)
Meet general needs	112 (22.0)	59 (23.6)	32 (17.2)	21 (28.8)
Almost enough to meet the needs	174 (34.2)	83 (33.2)	63 (33.9)	28 (38.4)
Fully meet the needs	42 (8.3)	21 (8.4)	18 (9.7)	3 (4.1)
Willingness to talk about sexual health^f^				
With parents/siblings	107 (21.0)	53 (21.2)	40 (21.5)	14 (19.2)
With intimate partner(s)	350 (68.8)	165 (66.0)	131 (70.4)	54 (74.0)
With community insiders/peers	284 (55.8)	102 (40.8)	134 (72.0)	48 (65.8)
With community outsider	87 (17.1)	18 (7.2)	46 (24.7)	23 (31.5)
With friends met online	131 (25.7)	24 (9.6)	78 (41.9)	29 (39.7)
With psychologist	129 (25.3)	48 (19.2)	56 (30.1)	25 (34.2)
With health manager	95 (18.7)	40 (16.0)	39 (21.0)	16 (21.9)
With healthcare staff	196 (38.5)	87 (34.8)	81 (43.5)	28 (38.4)
Unwilling to talk sex or rarely talk sex	15 (2.9)	7 (2.8)	4 (2.2)	4 (5.5)
Specific sexual knowledge needs^f^				
Female sexual health knowledge	370 (72.7)	188 (75.2)	133 (71.5)	49 (67.1)
Female guide to sexual pleasure	322 (63.3)	138 (55.2)	139 (74.7)	45 (61.6)
Intimate relationship instruction	354 (69.5)	156 (62.4)	144 (77.4)	54 (74.0)
Safer sex guidance	298 (58.5)	136 (54.4)	118 (63.4)	44 (60.3)

^a^ “Cis-heterosexual” group refers to cisgender heterosexual women.

^b^ “Sexual minority” group refers to cisgender sexual orientation minority women.

^c^ “Trans/nonbinary” group refers to transgender women and/or individuals assigned female at birth while identify as nonbinary gender.

^d^ 95% Confidence Interval for Mean.

^e^ All *p* values were reported by conducting comparisons using one-way ANOVA.

^f^ Participants could select more than one option.

#### Sexual behaviours of participants who had sexual experiences with a partner

Table 4 displays the specific sexual behaviours of participants who had sexual experiences with their partners (*N* = 336). Regarding the preferred mode of sexual behaviour, all three groups showed a strong preference for foreplay (84.1% in cis-heterosexual women, 87.6% in sexual minority women, and 74.0% in trans/nonbinary individuals). In terms of safer sex behaviours, consistent condom use was more prevalent among cis-heterosexual women. In contrast, sexual minority participants, reflecting the diversity of their sexual practices, more often adopted women-controlled safer sex strategies, such as self-cleaning before sex and cleaning of partners.

**Table 4.** Sexual behaviours of participants who had sexual experiences with a partner (*N* = 336)

Characteristics, *n* (%)	Overall (*N* = 336)	Cis-heterosexual^a^ (*n* = 157)	Sexual minority^b^ (*n* = 129)	Trans/nonbinary^c^ (*n* = 50)
First sex experience				
First time sex with a Female	93 (27.7)	1 (0.6)	62 (48.1)	30 (60.0)
First time sex with a Male	243 (72.3)	156 (99.4)	67 (51.9)	20 (40.0)
Penetrative sex ever				
Had penetrative sex before	292 (57.4)	138 (55.2)	113 (60.8)	41 (56.2)
Had non-penetrative sex before	44 (8.6)	19 (7.6)	16 (8.6)	9 (12.3)
Total lifetime sex partners, *M* [95%CI]^d^	3.4 [3.0 3.8]	2.3 [1.9 2.8]	3.9 [3.3 4.5]	5.2 [3.6 6.8]
Group sex				
Never	329 (97.9)	157 (100.0)	123 (95.3)	49 (98.0)
Had before	7 (2.1)	0	6 (4.7)	1 (2.0)
Sex trade				
Never	332 (98.8)	157 (100.0)	127 (98.4)	48 (96.0)
Had before	4 (1.2)	0	2 (1.6)	2 (4.0)
Preferred sexual behaviour^e^				
Foreplay	282 (83.9)	132 (84.1)	113 (87.6)	37 (74.0)
Oral sex	146 (43.5)	47 (29.9)	72 (55.8)	27 (54.0)
Finger sex	142 (42.3)	42 (26.8)	76 (58.9)	24 (48.0)
Masturbation	132 (39.3)	41 (26.1)	66 (51.2)	25 (50.0)
Vagina sex	193 (57.4)	109 (69.4)	62 (48.1)	22 (44.0)
Anal sex	15 (4.5)	1 (0.6)	7 (5.4)	7 (14.0)
Sex toys	109 (32.4)	21 (13.4)	62 (48.1)	26 (52.0)
Prefer other types	14 (4.2)	2 (1.3)	9 (7.0)	3 (6.0)
Ways to perform safer sex^e^				
Condom use	274 (81.5)	142 (90.4)	100 (77.5)	32 (64.0)
Self-cleaning	98 (29.2)	25 (15.9)	54 (41.9)	19 (38.0)
Clean sexual partner	192 (57.1)	61 (38.9)	100 (77.5)	31 (62.0)
Did nothing	21 (6.3)	8 (5.1)	8 (6.2)	5 (10.0)
Other ways for safer sex	10 (3.0)	7 (4.5)	2 (1.6)	1 (2.0)
Condom type often used^e^				
Common condom	243 (72.3)	143 (91.1)	70 (54.3)	30 (60.0)
Female condom	11 (3.3)	2 (1.3)	7 (5.4)	2 (4.0)
Finger condom	69 (20.5)	4 (2.5)	46 (35.7)	19 (38.0)
Oral condom	7 (2.1)	1 (0.6)	5 (3.9)	1 (2.0)
Other types	2 (0.6)	0	2 (1.6)	0
Never used	42 (12.5)	10 (6.4)	24 (18.6)	8 (16.0)
Condom use in the last 6 months (*n* = 237)				
Consistent use (Always use)	116 (48.9)	69 (60.5)	35 (38.9)	12 (36.4)
Usually use (More than half the time)	39 (16.5)	17 (14.9)	17 (18.9)	5 (15.2)
Seldom use (Less than half the time)	33 (13.9)	13 (11.4)	13 (14.4)	7 (21.2)
Never use	49 (20.7)	15 (13.2)	25 (27.8)	9 (27.3)

^a^ “Cis-heterosexual” group refers to cisgender heterosexual women (*n* = 157), and the percentages for this column were all calculated with 157 as the denominator.

^b^ “Sexual minority” group refers to cisgender sexual orientation minority women (*n* = 129), and the percentages for this column were all calculated with 129 as the denominator.

^c^ “Trans/nonbinary” group refers to transgender women and/or individuals assigned female at birth while identify as nonbinary gender (*n* = 50), and the percentages for this column were all calculated with 50 as the denominator.

^d^ 95% confidence interval for mean.

^e^ Participants could select more than one option.

### Qualitative findings

A total of 199 participants indicated their willingness to take part in interviews and provided contact information. To ensure diversity across gender identity, sexual orientation, age, place of birth, religion, occupation, and education level, 33 participants were recruited for follow-up interviews. The characteristics of these participants are summarised in Table 5, including 5 (15.2%) cis-heterosexual women, 18 (54.5%) sexual minority women, 2 transgender women, and 8 nonbinary individuals. Interviewees not only varied in their gender identity and sexual orientation, but also in age, education, employment, and relationship status. Their ages ranged from 20 to 38 years, with a mean age of 27.9 years (95%CI: [26.4, 29.5]).

**Table 5.** Demographic information for interview participants (*N* = 33)

Characteristics	*n* (%)
**Sex assigned at birth**	
Female	28 (84.8)
Male	4 (12.1)
Other (Unspecified genotype)	1 (3.0)
**Gender identity**	
Cisgender women	23 (69.7)
Transgender women	2 (6.1)
Gender queer	5 (15.2)
Gender fluid	1 (3.0)
Gender questioning	2 (6.1)
**Sexual orientation**	
Heterosexual	5 (15.2)
Homosexual	8 (24.2)
Bisexual	5 (15.2)
Pansexual	9 (27.3)
Asexual	2 (6.1)
Fluid or questioning	4 (12.1)
**Age (years)**	
Mean (95%CI)	27.9 (26.4 29.5)
**Age group**	
20-24 y	7 (21.2)
25-29 y	13 (39.4)
30-34 y	11 (33.3)
35-38 y	2 (6.1)
**Educational level**	
High school or below	2 (6.1)
College or undergraduate	23 (69.7)
Postgraduate and above	8 (24.2)
**Ethnicity**	
Han people	27 (81.8)
Ethnic minorities (Muslim, etc)	6 (18.2)
**Employment status**	
Full-time student	9 (27.3)
Full-time employed	15 (45.5)
Unemployed	6 (18.2)
Others (Freelancer)	3 (9.1)
**Migration situation**	
Local resident	12 (36.4)
Migrant	21 (63.6)
**Relationship**	
Single	9 (27.3)
Married (heterosexual marriage)	5 (15.2)
Divorced; have a stable partner	3 (9.1)
In a stable relationship	13 (39.4)
Polyamory or in a casual relationship	3 (9.1)

Overall, participants reported a range of perspectives on sexual health and sexual function, their own experiences of sex behaviours, as well as their perceived sexual pleasure. Four overarching themes were constructed in the analysis: (1) women’s sexuality has long been invisible but is changing; (2) diverse sexual identities and complex sexual practices; (3) the bond between sex and self-worth; and (4) disparities in safer sex awareness and practice.

#### Theme 1: women’s sexuality has long been invisible, but is changing

The long-standing invisibility of women’s sexuality is a common concern expressed by our interviewees, including conservative traditional female sexual scripts, deep-rooted negative views towards female sexual desire, and unheard female voices in sexual interactions under the male discourse on sexuality. For instance, a 31-year-old homosexual cisgender woman (ID2) reflected on the influence of her upbringing and articulated her internal conflict underlying her decision to enter into a traditional heterosexual marriage. She stated:

*“My family, although situated in a modern era, still retained certain patriarchal characteristics. My father, in particular, tended to be somewhat controlling and dominant, so I often found myself in a position of compliance. Consequently, I entered into marriage with my husband largely out of a sense of obedience. … However, given my mindset and the circumstances at the time, I felt that I had no alternative. … I feel like I am a pure lesbian and [the reason why] I entered into a (heterosexual) marriage was … to be loved, and [I] may be purely seeking a sense of security of being loved. … *”

Such narratives illustrate that, within a heteronormative and patriarchal social context, sexual minority women encounter multifaceted challenges, such as sociocultural expectations, familial pressures, and restrictive gender norms, which often result in the suppression of their authentic feelings. Similarly, there were cis-heterosexual participants who did not understand their desires but were unconsciously hiding and suppressing them. There were also heterosexual married women participants who reported experiencing conflicting emotions regarding extramarital affairs and one-night stands, simultaneously feeling a sense of liberation and internalised shame. For example, a 33-year-old cis-heterosexual woman (ID32), despite her intellectual clarity and autonomy as a scientist, struggled to describe her sexual desires. She reflected:

*“I sometimes question if I am bound by the social norms, and I often reflect on whether the education I received from childhood has overly restricted me in this regard [confronting sexual desire] … Anyway, I don’t think I am someone who particularly desires this thing [sex]; perhaps, deep down, I do not want to be someone who strongly desires it. Hmm … yes. It feels as though it is not that I lack this thing [sexual desire] entirely - it may still exist - but my mind, or perhaps subconscious repression, indicates me that I don’t want to acknowledge it. As a result, I conceal this part [my desire] of myself, hide it, and ultimately cannot see it [feel it].”*

Despite persistent challenges, the woman-specific sexual discourse is gradually taking shape, and women’s sexuality is also developing in the undercurrents. This progressive shift towards greater sexual agency spans across generations, with both younger and middle-aged women actively experiencing and expressing their sexuality in their own ways. For example, a 38-year-old cis-heterosexual woman (ID21) remarked, “*Sexuality is in every aspect of my life. It’s not just about love and responsibility. It is all-encompassing and fills my body, heart, and mind”.* Moreover, younger participants exhibit even greater openness and candour when discussing sexual matters. For instance, a 20-year-old pansexual cisgender woman (ID31) described a markedly different social environment, both for herself and her peers:

*“First of all, I’m sure that I do not experience sexual repression [laughs]. In fact, my high school environment was quite unconventional. I had sexual intercourse in some weird places at our school [laughs]. I think my friends are also very exaggerated (open), we always discussed topics such as sex, sexual fetishes, and our own sexual experiences without hesitation. We all feel that this [sex] is a very good thing, so in our group, I do not think anyone, whether boys or girls, felt sexually repressed.”*

Embracing diversity is crucial for achieving a more comprehensive and inclusive understanding of women’s sexuality. For example, a 36-year-old transgender pansexual woman (ID12) shared her personal experience, which helps illuminate the often-overlooked sexual needs present at the margins:

*“For many of us [trans women], there’s a slight [anatomical] difference, just a small difference in scale, compared to cisgender women, while this small difference can sometimes cause problems. For me, using my own fingers for vaginal masturbation is definitely not an issue. But when it comes to buying some tools [sex toys] online, I often find that the sizes don’t match. Nowadays, there are some small toys designed specifically for women - not those particularly large, male-oriented ones [like dildos]. For example, some toys can stimulate both the vagina and the clitoris at the same time - you must know what I mean, right? [Interviewer: Sure] But for me, a transwoman, it’s often impossible to satisfy both areas at once. If it reaches my vagina, it can’t reach my clitoris; if it reaches the clitoris, then the bottom half is left outside [sighs].”*

Women are increasingly speaking out about their sexual experiences and desires, contributing to a shift towards a more woman-centred sexual discourse. This transformation has the potential to help break down barriers that have long hindered women’s sexual liberation and pave the way for a more equitable understanding of sexuality. While there is a growing recognition that women’s sexual health and well-being are important, equally important is the need to ensure that the voices and specific needs of all women are acknowledged – particularly those of women from gender minority groups, whose embodied experiences are often more complex and nuanced.

#### Theme 2: diverse sexual identities and complex sexual practices

The relationship between the plurality of sexual identities and the diversity of sexual practices is complex, shaped by the intersection of social identities and individual lived experiences. On one hand, the exploration and confirmation of one’s sexual identity may encourage various sexual behaviour experiments. For example, a 24-year-old homosexual cisgender woman (ID22) shared how she and her partner frequently engage in a variety of sexual activities, saying that:

*“We have a relatively high level of sexual satisfaction, that is, we try a lot of different [forms of sexual activity]. I think it is quite common, since we are already lesbians, are already sexual minorities, already social niche. For this reason, I don’t think it’s necessary to conform to prescriptive (or normative) sexual behaviours”*.

This account illustrates how occupying a marginalised sexual identity can foster a sense of freedom from normative expectations, thereby enabling more diverse sexual practices. Similarly, an interviewee in a mixed-gender relationship (a 30-year-old bisexual cisgender woman, ID23), after sharing her exploration of sexual orientation, also took the initiative to discuss the sexual preferences she and her partner enjoy:

*“We started playing the Fourth Love [Pegging] because he seemed interested in women’s clothing. He bought some feminine, sexy outfits for himself - high heels, skirts, and stockings - and would wear them during our encounters. We both know he is not gay, he just liked to dress that way and he only did so when it’s just the two of us. As time went on, I came to accept this part of him, and I genuinely complimented him on how beautiful he looked in women’s clothes. I felt that, for him, this represented a complete sense of sexual liberation.”*

Other self-oriented empowering sexual behaviours, such as different forms of masturbation or unconventional attempts to achieve sexual pleasure, were noted by interviewees with different sexual identities. While survey data indicated that trans/nonbinary participants reported lower levels of sexual satisfaction and positive sexuality, the transgender women interviewed in this study (all of whom were undergoing or had completed gender-affirming surgery) expressed that, aside from unavoidable physiological differences, they perceived little distinction between themselves and cisgender women. For example, a 32-year-old homosexual transgender woman (ID1) shared:

*“Like us transgender women, there is no way to automatically secrete lubrication [natural lubrication], so we all need to use lubricants during sex. But other than that, our sex lives are actually not much different from those normal [general] ones, typically those between a man and a woman.”*

On the other hand, despite having engaged in a variety of sexual experiences, some participants continued to express uncertainty and a sense of ongoing exploration regarding their sexual identities, intimate relationships, desires, orgasms, and other forms of pleasure. For example, a 23-year-old participant (ID4), who identifies as both lesbian and queer, shared her sexual concerns and described moments of uncertainty and ambivalence in her intimate experiences. She reflected on her partner’s sexual pleasure and her own sense of confusion:

*“What left a deep impression on me actually wasn’t my own experience, but rather situations where my partner seemed to feel very happy. For example, she would, hmm … , reach hmm … [Interviewer: Orgasm?] Yes! Multiple orgasms, and sometimes things like … squirting, or even some incontinence. But I often feel confused - when I see my partner enjoying herself so much, I don’t really understand why she feels that much pleasure. On the other hand, I even wonder, hmm … why does she feel so good, but when I am being penetrated, I never have that same feeling? I just feel confused about that … [several long pauses in between]”*

These narratives suggest that sexual behaviours are shaped not only by sexual identity but also by the interplay of socioeconomic background and relational contexts. The diversity of sexual practices among participants highlights the importance of acknowledging the multiplicity and intersectionality of sexual and gender identities, as well as the diverse realities they produce.

#### Theme 3: bond between sex and self-worth

Interviewees almost universally rejected the view that female sexual function is primarily for reproduction. Instead, they interpreted it as multidimensional and highly self-relevant, inseparable from relationships. Although there were no observed differences in sexual function levels among the three sexual identity groups, correlational analysis revealed a positive association between sexual function and self-esteem (Supplemental Table S1). Additionally, higher self-esteem was also linked to more positive sexuality and greater sexual satisfaction. These quantitative findings are vividly illustrated by qualitative interviews, such as that of a 36-year-old transgender pansexual woman (ID12), who expressed a deep love for her body and herself, reflecting:

*“I’m not sure how to explain this - what is good sexual function? To me, it’s just that everyone feels ‘Shuang’ [pleasure], that’s what makes it good! [Interviewer: So … what does ‘Shuang’ mean?] For me, ‘Shuang’ means being able to reach orgasm. To be more specific, during orgasm I might experience fluid discharge, some spasms or twitching, and a sensation I’d describe as a ‘brain orgasm’ - a comfortable and refreshing feeling.”*

Overall, participants placed significant value on both sexual pleasure and spiritual fulfilment, yet expressed ambivalent feelings about orgasm. The struggle of faking orgasm emerged as a recurring challenge, reflecting the intricate relationship between sexual fulfilment and self-perception. Some participants, for instance, only came to believe in the existence of orgasm after experiencing it firsthand. As a 29-year-old homosexual cisgender woman (ID8) shared,

*“I always thought that orgasm was something like a ghost or something I had never seen until I experienced it once. Because my partner [at that time] was more skilled at that, so now I basically have orgasm(s) every time, and the squirting also appears often”.*

In contrast, others remained doubtful about the existence of vaginal orgasm, such as a 33-year-old cis-heterosexual woman (ID32) stating that, “*I don’t think it exists, and even if others claim it exists, I very much question the existence of this thing*”.

These diverse perspectives and experiences of orgasm in intimate encounters often influenced participants’ self-perception and confidence. Interestingly, correlational analysis indicates that psychological well-being, such as self-esteem, was positively linked to orgasm but not to desire (Supplemental Table S1). This might be explained by the mind–body connection involved in the perception of sexual pleasure. For instance, a 24-year-old homosexual transgender woman (ID20) described a profound transformation in her relationship with her body and her ability to experience sexual pleasure after undergoing gender-affirming surgery:

*“When I was in a male body, I could ejaculate, but the sensations afterward just … made me feel uncomfortable. I never experienced orgasm or any real pleasure. It was only after I had the surgery and my body have been updated that I was finally able to feel sexual pleasure and orgasm.”*

Together, these accounts illustrate an intuitive connection between self-perceived sexual function and self-worth, highlighting how self-perception can enhance one’s capacity for sexual fulfilment. Meanwhile, participants with different sexual identities shared similar experiences of feeling sexually objectified, particularly in the context of unenjoyable or painful sexual experiences. This objectification often led to a sense of diminished self-worth and negative emotions such as shame and guilt. For instance, a 23-year-old pansexual cisgender woman (ID28) recounted,

*“I felt it was unfair, I didn’t want to have any sexual intercourse - it’s unfair, why am I the only one in pain and you (her partner) aren’t? [Her voice started to sound a little choked up, then she cried for a while before calming down] … I would be very sensitive to the pain when he penetrated, but he just didn’t understand. … I started to feel as if there was something wrong with me. … During that time, I was very resistant to sex, and I interpreted my boyfriend’s sexual invitations as meaning that I was only valuable to him for sex, and that was all he cared about.”*

Similarly, a 29-year-old cis-heterosexual woman (ID27) described her struggle with vaginismus:

*“When I found this problem, I went to the hospital for examination, and after checking twice I was told that I had no problem, but it still hurt when it [his penis] went in [my vagina]. I tried to use some tool [vaginal dilator] slowly, little by little, slowly letting it [my vagina] expand and expand, and then after a while, he could come in. BUT, but I found that I didn’t have any pleasure inside, that feeling is very uncomfortable, both physically and psychologically. It just feels like I am a pile of meat on the chopping board, and then constantly being stabbed by a knife, again and again … I feel the vaginal penetration is mainly to satisfy male desires, not to satisfy my desires, there is no pleasure, only pain … ”*

These accounts clearly illustrate how negative sexual experiences can undermine a person’s sense of self-worth and self-esteem, both of which are crucial to women’s sexual health and overall well-being. When these qualitative narratives are considered alongside the quantitative findings, a subtle but important complexity emerges. For example, cis-heterosexual women reported lower levels of sexual desire and less pain compared to both minority groups,^29^ but in interviews, women in heterosexual relationships frequently expressed strong emotions related to pain. This contradiction may be influenced by social desirability, as people in mainstream relationships might downplay pain or desire to fit in, leading to underreporting in surveys. In contrast, qualitative interviews help reveal deeper, more authentic experiences that surveys may overlook. Despite these differences, gender inequality in sexual experiences remains a serious issue. It is essential to recognise and address all forms of pain and distress in sexual relationships, ensuring that each individual’s suffering is acknowledged.

#### Theme 4: disparities in safer sex awareness and practice

Our interviewees demonstrated a relatively good understanding of safer sex, but there were noticeable discrepancies between their awareness and actual practices. This gap was evident in the regret some participants expressed about their past decisions. For example, a 31-year-old cis-heterosexual woman (ID25) admitted, “*In fact, I know the risk [of getting infected with STIs] could be very high, but at that time, I felt that I was afraid of troubling others (to wear condoms); … anyway, forget it [alas] … *” There were also others who were surprised to realise the disconnect between what they knew and how they acted, having never previously considered the reasons behind their choices. For instance, a 30-year-old bisexual cisgender woman (ID30) who had experienced an abortion shared,

*“I know it is definitely necessary to wear a condom when having sex, either for contraception or disease prevention, it is certainly the best. But I always feel, umm, I have a sense of fluke. … You know what, I always prepare the pregnancy test at home, and I use it once a month on average, which is more frequent than condom use [laughs], and even if I know it’s going to be fine [won’t be pregnant], I still want to take a test, it’s kinda weird, I don’t know what I am thinking [laughs] … ”*

Some participants described feeling powerless to protect themselves, further highlighting the complexity of safer sex behaviours. A 20-year-old pansexual cisgender woman (ID31) recounted a particularly distressing experience:

*“I once had sex with someone I considered relatively high-risk, though I realize that ‘high-risk’ can be hard to define. Anyway, that was a really high-risk sex with a man who has a lot of tattoos, and he told me that he also used drugs, though I’m not sure if that involved needles. But when we were having sex, he unexpectedly punctured the condom because he wanted to force me into a relationship. I didn’t agree, so he deliberately damaged the condom. I was really frightened and took preventive pills several times afterward. Even so, I remained scared and went to the hospital for the HIV test, which thankfully came back negative. After that, I kept testing it once a month, every month. Since I can use my menstrual blood for the tests, I did the testing every month regularly. But [I] haven’t tested recently, so now I’m starting to feel anxious again … ”*

In contrast, some interviewees, particularly those in same-gender relationships, described more proactive approaches to safer sex. For example, a 22-year-old homosexual cisgender woman (ID24) shared:

*“Soon after we got together, we both went to get checked for diseases related to sex [STIs]. Given that our female bodies are more fragile and may be easily infected by bacteria or germs, like if the mucous membranes get torn, so we usually pay attention to self-protection. For example, she used to like manicures, but now she doesn’t do it.”*

Similarly, while continuously exploring non-mainstream sexual practices such as bondage, discipline, dominance, submission, sadism, and masochism (BDSM), some participants remained proactive in practising sexual safety, even though they still experienced unclear bodily sensations and some confusion regarding desire and orgasm. As a 23-year-old homosexual cisgender woman/queer (ID4) emphasised,

*“We pay a lot of attention to things like using finger condoms, cleaning any tools or toys we use, and also making sure the intensity is appropriate. Like, what level is … um … not dangerous. Because we have tried BDSM many times, I know there can be a lot of accidents. So anything I want to use on my partner, I always try on myself first, and only use it on her if there is no problem. For me, personally, I actually know quite a lot about sexual safety … but sexual pleasure is something I still lack.”*

However, the promotion of safer sex relies not only on individual willingness or behaviour, but is also constrained by the accessibility and quality of available products. Even among those who are motivated to practice safer sex, practical barriers sometimes hinder consistent use. For example, a 36-year-old transgender pansexual woman (ID12), described her experience using protective products:

*“My partner is a transgender man. Since we’re at home together every day, our usual routine for safer sex is just to wash our hands and use condoms. [Interviewer: What kind of condoms do you use specifically?] Just regular condoms for the dildo. We’ve also tried dental dams for oral sex, but honestly, the taste is really unpleasant, awful, weird, and greasy in texture. Although most are fruit-flavored, the artificial scent is so overpowering, like the kind you’d find in the cheapest popsicles, the ones you would never want to eat. The combination of artificial fragrance and oily odor is so strong and synthetic that it’s nauseating … ”*

These accounts collectively highlight that participants’ actual sexual behaviours are shaped by a complex interplay of relational pressures, personal agency, emotions, and practical barriers. The attributes of the relationship also matter, for those in stable, mutually trusting same-gender relationships, or in mixed-gender relationships without a need for contraception, the relationship itself can provide a sense of sexual safety, making condom use less necessary. These qualitative findings help explain the differences in condom use observed in the survey; inconsistent condom use does not necessarily mean that individuals are not practising safer sex. Instead, issues like uncomfortable condom design and negative experiences can discourage regular use, suggesting that future interventions should address not only knowledge, but also the broader social, relational, and product-related factors that influence safer sex behaviours.

## Discussion

Sexuality is deeply embedded within the sociocultural context in which individuals live. In China, the rapid expansion of Internet access and social media platforms has exposed younger generations to more sex-related materials, gradually weakening the hold of traditional norms.^21,74^ Although society is often perceived as becoming more sexually open,^18^ traditional female sexual scripts still significantly shape the sexuality of Chinese women. Therefore, contemporary Chinese sexual culture is often described as *dispersive and colourful*,^75^ marked by a complex coexistence of freedom and conservatism, liberation and repression, inclusivity and exclusion, as well as respect and discrimination. Within this dynamic environment, our study is particularly important, as it sheds light on how broader sociocultural trends shape women’s sexuality.

This is the first study in China to comprehensively investigate sexual health and sexual behaviours among women with diverse sexual identities using both quantitative online surveys and qualitative in-depth interviews. Drawing on the sexual health model^38^ and the intersectionality lens,^55^ our findings demonstrate that sexual identity has suggestive associations with various aspects of sexual health, sexual function, sexual perceptions, sexual experiences, safer sex behaviours, and sexual well-being. However, sexual identity is just one aspect of an individual, and other factors such as age, education, ethnicity, and upbringing environment also play a role in shaping one’s own sexual script.

The average age of first sexual intercourse among our participants (20.4 years) matches that reported in the 2015 Chinese national survey.^18^ When examining sexual identity exploration, cis-heterosexual women reported earlier identity confirmation but later sexual debut compared to their minority counterparts, a pattern consistent with international research.^76,77^ This may be because cisgender heterosexual identity aligns with societal expectations, making self-recognition easier, while ongoing taboos around sexuality can foster unrecognised repression and shame, delaying sexual initiation. In contrast, sexual minority women often explore their identities through sexual practice, which may lead to earlier sexual activity but later identity confirmation. Consistent with previous research,^14,78^ our quantitative and qualitative findings integrally highlight the diversity of sexual experiences corresponding with the plurality of sexual identities, and suggest that the diversity of sexual identities may facilitate the exploration of sexual practices and expand possibilities for intimate relationships.

Our findings (supplemental tables) also indicate that social and cultural factors may influence the timing of sexual debut and sexual identity development. Participants from more economically developed regions reported earlier sexual initiation, while those with higher education or stable employment tended to delay it. Ethnic minority women reported a later onset of both masturbation and intercourse, in line with previous studies.^18,79^ Although no obvious associations were found between ethnicity, education, or birthplace and the timing of sexual identity recognition, migration status was linked to the age of identity confirmation. Increased population mobility has contributed to a more pluralistic cultural landscape in mainland China, similar to the trends observed in Hong Kong,^80^ suggesting that exposure to diverse cultural environments can shape one’s sexual values and attitudes.

When focusing on sexual health outcomes, cis-heterosexual women reported noticeably higher sexual satisfaction and more positive sexuality. This may reflect the psychological comfort of aligning with societal norms, but could also indicate a tendency to conform to social expectations, such as faking orgasm. Interestingly, despite higher satisfaction, heterosexual women reported lower sexual desire than non-heterosexual women,^29^ underscoring the subtle, enduring influence of sexual conservatism. Gender minority participants (e.g. transgender women) reported more negative sexual attitudes and satisfaction, likely due to the unique challenges of exploring their relationship with their bodies. In addition, our validation findings demonstrated that sexual minority women have a higher prevalence of potential sexual dysfunction and notably lower sexual satisfaction compared to cis-heterosexual women.^29,59^ These findings highlight the need for future interventions and policies to address the unique sexual health needs of this population.

Another notable finding is that foreplay was ranked as the most preferred sexual activity across all groups, echoing participants’ desire to rename it as “mainplay”. That is, activities such as kissing and caressing were reported to provide women with more pleasure than penetrative sex, suggesting that these acts should not be considered merely as foreplay, but rather as a central and complete part of the sexual act. These findings underscore that positive sexual exploration is crucial for women’s sexual health and well-being, and highlight the need to develop woman-centred sexual discourse and create sexual scripts on women’s own terms.

Regarding safer sex practices and perceptions, sexual minority women report relatively more lifetime sexual partners, greater engagement in group sex, online hookups, non-monogamous relationships, and increased substance use compared to cis-heterosexual women. While these behaviours are often labelled as “risky” or “unsafe” due to their association with increased STI transmission, such risk-based language can perpetuate stigma and hinder sexual health promotion.^81^ However, as condom needs vary across different sexual relationships, sexual minority women practised a greater variety of safer sex behaviours, such as frequent clean-before-sex routines and relatively common use of finger condoms. Our in-depth interviews further revealed their diverse understandings of intimacy and sexual safety, with participants emphasising that safer sex is not always linked to condom use. While sexual minority women demonstrated agency in adopting safer sex practices, they frequently voiced concerns about the lack of tailored sexual health content, indicating the challenges of designing effective safer sex programmes to address their unique needs. There is an urgent need for future research to establish tailored safer sex guidelines for these populations and to ensure that this information is widely accessible. As previous studies suggest,^14^ tailored interventions that address the unique experiences and needs of sexually diverse individuals may be more effective in promoting safer sex practices.

Despite increasing exposure to sexual materials among Chinese young women,^18^ sexuality education in China is often inadequate or absent.^82^ Access to sexual and reproductive health information and services also remains uneven, with rural women, ethnic minorities, and migrants facing greater obstacles.^79^ In our study, only 8.3% of participants felt their current sexual health information needs were fully met, and disparities existed across groups. Over half of cis-heterosexual women had never been exposed to information about female sexuality, either due to lack of access (27.6%) or not knowing where to find it (25.6%), indicating substantial unmet needs. For gender and sexual minority women, although they actively seek to explore their sexuality, research in Western contexts suggests that their minority status may limit the social resources available to them.^83^ This is partly because healthcare providers and systems are perceived as heteronormative, which may discourage them from seeking medical services.^16^ Future interventions should move beyond traditional condom promotion to address the specific practices and needs of diverse groups.

Most participants expressed the need for women-specific sexual health knowledge, with sexual and gender minority participants reporting more willingness to seek tailored guidance on female sexual pleasure and intimate relationships. Qualitative interviews further confirmed that access to diverse or personalised sexual knowledge can help women better explore and understand themselves. Many also emphasised the importance of peer support in their sexuality development, noting that being able to talk openly about sex with peers was crucial. These findings suggest that sexuality researchers and service providers need to fully consider individual identity, sexual values, and lived realities in order to offer more targeted and effective information and support.

Clear women-centred language about female sexuality is crucial for women’s self-understanding. For example, one participant (ID23) who practised the *Fourth love* once doubted her partner’s sexual orientation and her own self-worth before learning about this concept. By accessing relevant information and personally experiencing this non-mainstream sexual practice, she gained a deeper understanding of herself, her partner, and their relationship. However, many participants struggled to recognise or articulate their most authentic thoughts. These complex feelings mirror the theme of *cultural unintelligibility* noted in previous research on Chinese minority women.^32^ Although Chinese society is evolving, ongoing tensions between modernity and tradition, agency and authority, as well as openness and censorship, continue to shape personal sexual values. Our in-depth interviews provided a safe space for participants to express their desires, share sexual experiences, and challenge sexual norms, contributing to fostering a positive and empowering view of female sexuality.

Sexuality is a fundamental aspect of daily life, and sexual practices are not merely tools but rather practices that create meaning and shape the purpose of life.^84^ However, our study highlights the psychological challenges faced by sexual orientation and gender minority women during identity exploration. More than half of gender minorities and about one-third of sexual orientation minorities reported experiencing psychological distress during identification, underscoring the need for psychosocial support. Unlike countries where same-sex marriage is legal, sexual minority populations remain relatively invisible in China. These findings emphasise the importance of considering both sociocultural context and individual identity development in understanding and supporting Chinese women’s sexual health and well-being. Thus, the collective contributions of medical professionals, social service providers, and multidisciplinary experts in the field of sexuality are highly needed.

To our knowledge, this study is the first in China to comprehensively examine sexual health and behaviours among women of diverse sexual identities, addressing important gaps in research on non-Western and marginalised populations. Using a sequential mixed-methods design and an intersectional approach, we provide a nuanced understanding of how individual identity, social status, and cultural context shape women’s sexuality and sexual health. The findings reveal that, beyond individual knowledge or attitudes, broader cultural and structural factors, such as a lack of culturally relevant sexual health resources and persistent social stigma, may create practical barriers to consistent safer sex practices, even for well-informed women. Another major strength of this study is the use of reflexive thematic analysis as our qualitative analysis method,^69^ which not only fully appreciates the subjective value of the interviewer but also allows the analysis to be iterative and reﬂexive. This method allows different stages of analysis to be looped back and forth, making the presentation of results richer and more nuanced.^85,86^

This study has several limitations. First, due to practical constraints and the sensitive nature of the topic, we employed non-probability sampling, resulting in a sample predominantly composed of young adults. This may limit the generalisability of our findings to all Chinese women. Future research should consider adopting random sampling methods to include a more diverse sample, particularly women from older age groups and individuals from other sexual minority groups, in order to obtain a broader and more comprehensive understanding of this population’s experiences and insights. Second, the present study did not conduct further subgroup analyses, and differences between individuals of varied sexual orientations and different gender identities require further analysis in larger, more diverse, and representative samples. Third, although the in-depth interviews are generally considered to reduce social desirability bias, the questionnaire responses are still likely to be affected,^43^ especially among participants whose identities or experiences align with mainstream norms. Future research should include measures of social desirability when asking sexually sensitive questions and consider this factor during data analysis. Lastly, all data were collected online, potentially excluding individuals without Internet access and resulting in a relatively younger sample in this study. In the future, more diversified approaches, such as face-to-face interviews, could be considered to ensure a more representative sample.

It also needs to be acknowledged that this study was conducted during the global COVID-19 pandemic. The associated psychosocial impacts and quarantine measures may have affected participants’ sexual health and behaviours, particularly among women.^87^ Consequently, ongoing surveys are essential to better understand the evolving sexual health needs of women with diverse sexual identities. Despite these limitations, this study provides valuable insights into the sexual health and behaviours of sexually and gender-diverse women in China. Overall, our findings underscore the need for tailored, culturally sensitive, and identity-affirming interventions to support both identity development and sexual well-being.

## Supplementary Material

Questionnaire: Sexual health survey of women with diverse sexual identities.

Supplemental tables S1 and S2

## Data Availability

Quantitative data supporting the conclusions of this article are available from the first or corresponding author on reasonable request. While qualitative data will not be made available because it contains confidential individual information.

## Footnotes

* The sexual health model is anchored in a holistic deﬁnition of sexual health and provides a theoretical framework for improving people’s overall sexual well-being. The model deﬁnes 10 content components that constitute essential aspects of healthy human sexuality, including (1) Talking about sex, (2) Culture and sexual identity, (3) Sexual anatomy and functioning, (4) Sexual health care and safer sex, (5) Challenges to sexual health, (6) Body image, (7) Masturbation and fantasy, (8) Positive sexuality, (9) Intimacy and relationships, and (10) Spirituality.

† The concept of sexual well-being in the academic literature involves a wide range of aspects, and there is no unified definition. An operational model of sexual well-being was proposed, and the specific sexual well-being framework was conceptualised, which resonates with the biopsychosocial model. The framework links sexual well-being to sexual health, and relates it to two other pillars, sexual pleasure and sexual justice.

## Provenance statement

This article was not commissioned and went through external peer review.
