# Supplementary material for: Sexual health and sexual behaviours in Chinese women of varied sexual identities: a sequential mixed methods study
Source: Sex Reprod Health Matters. 2026 Feb 9;33(1):2624200. doi: 10.1080/26410397.2026.2624200 (PMC13112868; doi:10.1080/26410397.2026.2624200)
Supplement: Questionnaire: Sexual health survey of women with diverse sexual identities. [file ZRHM_A_2624200_SM9343.docx]

**Sexual health survey of women with diverse sexual identities**

**Part I Informed Consent Form**

You are invited to participate in a research study regarding the sexual health of women with diverse sexual identities.

**Purpose of the study**

The purpose of this study is to explore the health status and health needs of women, including both sexual minority women and heterosexual women.

**Procedures**

You will be invited to complete an online survey at the time of entry into the study, which takes approximately 30 minutes. After that, you will be invited to participate in an online interview, which takes around 30-60 minutes. You would be audiotaped during the interview after obtaining your consent.

**Potential risks**

Participating in this research activity does not pose any known danger to you.

**Confidentiality**

The information obtained during the study period will be kept absolutely confidential and will not be circulated to anyone outside the study group. Information that identifies you personally will be replaced using anonymous encoding, that is, the participant number without any personal identification.

**Participation and withdrawal**

Your participation in this study is voluntary and you are free to withdraw from this study at any time without necessarily giving a reason and without any legal rights being affected.

**Questions and concerns**

If you have any questions about the research, please feel free to contact **Chanchan (Tel: …; Email:** …). If you have questions about your rights as a research participant, contact the Human Research Ethics Committee, HKU (…).

**SIGNATURE**

I ______________________ (Name of Participant) understand the procedures described above and agree to participate in this study.

I agree to the audio-recording during the procedure. (If participating in the interviews)

- Yes
- No

HREC Approval date: [July 8, 2021]

HREC Reference Number: [EA210325]

**Part II Questions**

**Sociodemographic data**

Please fill out the form according to your situation and select or fill in the blanks with the option that best suits your situation.

1. Age: _______years old
2. What is your education level?

- Primary school and below
- Junior high school
- High school/secondary school
- College/Bachelor
- Graduate school and above

1. What is your ethnic identity?

- Han people
- Zhuang people
- Hui people
- Manchu
- Tibetan
- Others, please indicate_____________________________________

1. What is your current occupation? Or what do you do most of the time?

- Unemployed
- Retired
- Students (full-time)
- Organization officers
- Professional and technical personnel (research/teaching/medical/artist, etc.).
- Clerks and related personnel (administrative/police/firefighting, etc.)
- Life service personnel (food and beverage/sales/banking/real estate, etc.).
- Farmers/pastoralists/fishers/forestry production and support staff
- Manufacturing and related personnel (workers)
- Soldier
- For the other things, please explain_____________________________________

1. What is your monthly income (China Yuan, CNY)?

- No income (e.g. student/unemployed) or less than 1000 CNY
- 1001 to 3000 CNY
- 3001 to 5000 CNY
- 5001 to 7000 CNY
- 7001 to 9000 CNY
- 9001 to 11000 CNY
- Above 11000 CNY

**Sexuality identity**

*Self-identity may change over time, so please answer the following questions in relation to your current identity and past life experiences. There is no right or wrong answer, and all responses will be kept confidential, so please feel free to respond truthfully.*

***Gender identity***

1. What is your birth assigned gender?

- Female
- Male
- Intersex
- Others, please indicate_____________________________________

1. What is your official gender (gender on your ID card)?

- Female
- Male

1. What is your gender identity?

- Cisgender women
- Cisgender men (Questionnaire closed)
- Transgender women
- Transgender men (Questionnaire closed)
- Queer
- Gender fluid
- Unsure
- Others, please indicate_____________________________________

1. At what age have you confirmed your gender identity? (i.e., being aware of your gender)

At the age of _____________________

1. How have you felt since you knew your gender identity? (Multiple options available)

- Developed naturally and everything went well
- My sexual orientation makes me feel anxious sometimes
- My sexual orientation makes me feel depressed sometimes
- My sexual orientation makes me feel lonely sometimes
- I have had suicidal thoughts, but no suicides
- I have had both suicidal thoughts and attempted suicide

***Sexual orientation identity***

1. The people who attract you emotionally (spiritually) are:

- Women only
- Most are women
- Both women and men
- Most are men
- Men only
- I cannot tell

1. The people who attract you physically (sexually) are:

- Women only
- Most are women
- Both women and men
- Most are men
- Men only
- I cannot tell

1. What is your sexual orientation?

- Homosexual
- Bisexual
- Heterosexual
- Pansexual
- Not sure
- Others, please indicate_____________________________________

1. At what age have you confirmed your sexual orientation?

At the age of _____________________

1. How have you felt since you knew your sexual orientation? (Multiple options available)

- Developed naturally and everything went well
- My sexual orientation makes me feel anxious sometimes
- My sexual orientation makes me feel depressed sometimes
- My sexual orientation makes me feel lonely sometimes
- I have had suicidal thoughts, but no suicides
- I have had both suicidal thoughts and attempted suicide

If not cisgender heterosexual women, continue to answer the following questions:

1. How is your “coming out” status?

- Did not come out
- Not fully come out, only friends know
- Not fully come out, only family members know
- Not fully come out, most people know
- Fully come out
- Others, please indicate_____________________________________

1. What (sexual) role do you consider yourself in a female-female partnership?

- T (Tomboy, masculine-presenting)
- P (Pure girl/ Po-“wife”, feminine-presenting)
- H (Half, with a more androgynous gender style)
- No fixed roles
- Prefer not to be labeled
- Others, please indicate_____________________________________

1. Which title do you think you most like to accept?

- 拉拉 (Lala, a Chinese loose translation of lesbian)
- 蕾丝边 (Chinese equivalent of the word lesbian)
- 同性恋 (Homosexual)
- 同志 (Tongzhi, local identity of LGBTQ+)
- 女女性行为者 (Women who have sex with women, WSW)
- 女双性恋 (Bisexual women)
- 女泛性恋 (Pansexual women)
- Others, please indicate_____________________________________

1. Have you ever had a one-night stand via online dating (hookup online)?

- Yes, please indicate_____________________________________
- Never

**Intimate relationship**

1. What is your current intimate relationship situation?

- Single
- Unmarried, have a female partner
- Unmarried, have a male partner
- Unmarried, have a transgender partner
- Married (heterosexual marriage)
- Married (same-sex marriage), please indicate the specific form___________________
- Divorced
- Widowed
- Others, please indicate_____________________________________

1. What type of relationship are you in? (Would not display if last item answered “single”)

- We both adhere to monogamy
- I am monogamous, but my partner is not
- I am monogamous, but not know what view my partner holds
- My partner is monogamous, but I am not
- I am not monogamous, and not know what view my partner holds
- None of us practice monogamy
- Others, please indicate_____________________________________

1. What is your own view of marriage? (Multiple options available)

- Might into a heterosexual marriage
- Might marry a gay (fake marriage)
- Stand firm to wait the legal same-sex marriage
- Will never into a heterosexual marriage
- Might choose pre-determined guardianship
- No ideas yet
- Others, please indicate_____________________________________

**Sexual behavior and sexual health**

*The following questions are primarily about your sexual behaviors and sexual health, so it is recommended that you fill out the form in a safe and private environment. Please choose the answer that best suits your situation.*

***Sexual experiences***

1. Have you ever masturbated?

- Never had (jump to item 27)
- Yes

1. How old were you when you first masturbated?

At the age of _____________________

1. How often have you masturbated in the past year?

- Several times per day
- 1 time per day
- 1 to 3 times per week
- 1 to 3 times per month
- Once every 2~3 months
- Once every 6 months or less

1. Have you used sex toys to masturbate?

- Yes
- Never had

1. Have you had engaged in any other types of sexual behavior before?

- Never had sexual intercourse with any sex partners before (would skip NSSS-S scale)
- Only ever had sexual contact or other sexual behavior without penetrative sex
- Had penetrative or other sexual behaviors
- Others, please indicate_____________________________________

1. At what age did you have your first sexual encounter?

At the age of _____________________

1. What gender was your first sexual encounter with?

- Cisgender woman
  - Have you ever had sex with people with other gender identities?
    - No
    - Yes, with cisgender men
    - Yes, with transgender people, please indicate______________________
- Cisgender man
  - Have you ever had sex with people with other gender identities?
    - No
    - Yes, with cisgender women
    - Yes, with transgender people, please indicate______________________
- People with other gender identity, please indicate ___________________________
  - Have you ever had sex with people with other gender identities?
    - No
    - Yes, with cisgender women
    - Yes, with cisgender men
    - Yes, with other transgender people, please indicate___________________

1. What kind of sexual behavior do you prefer? (Multiple options available)

- Foreplay (kissing/touching)
- Oral sex (oral love)
- Hand/finger crossing
- Fisting
- Masturbation (self-exploration)
- Vaginal penetration
- Anal sex
- Sex toys
- All above
- Others, please indicate_____________________________________

1. Do you currently have (a) stable sexual partner(s)?

- Yes, with a man
- Yes, with a woman
- Have sexual partners, but not stable
- No sexual partner for the time being

1. How many different women have you had sex with so far?

_____________

- - Have you had sex with women in the last six months (oral, vaginal, anal sex, etc.)?
    - Yes, please indicate the number _____________
    - No

1. How many different men have you had sex with so far? _____________
   - Have you had sex with men in the last six months (oral, vaginal, anal sex, etc.)?
     - Yes, please indicate the number _____________
     - No

***Safer sex behaviors***

1. What method do you generally use for security protection? (Multiple options available)

- Use different types of condoms (if select yes, then move to the next)
- Clean your own hands/mouth/vagina/anus/sex toys, etc
- Clean both your and your partner’s hands/mouths/vaginas/anus, etc
- Never used any form of safer sex behaviors
- Others, please indicate_____________________________________

1. What type of condom do you often use? (Multiple options available)

- Never used, please indicate reason _____________________________
- Common condoms
- Female condom
- Finger condom (findom)
- Oral condom
- Others, please indicate_____________________________________

1. How do you usually get condoms? (Multiple options available)

- Offline supermarket/convenience store
- Offline pharmacies/sex health care stores/vending machines
- Collected for free
- Online shopping
- All prepared by partner.
- Others, please indicate_____________________________________

1. How often have you used condoms during penetration sex in the last six months?

- Use condoms every time
- Usually use (more than half the time)
- Seldom use (less than half the time)
- Never use

1. Did you use a condom the last time you had penetration sex?

- Yes, used condom
- Yes, used findom
- Yes, used oral condom
- Yes, used other type of condom, please indicate____________________________
- Did not use

1. If condoms are not used every time, what is the reason? (Multiple options available)

- No condoms available at that time
- I do not want to use it
- My partner(s) do not want to use it
- Feel no risk
- Cannot stand the smell of condoms
- Too expensive
- Condoms would influence the feeling (lack of pleasure)
- Others, please indicate_____________________________________

1. Have you had commercial sex ever?

- Never had
- Yes, had bought sexual services (you paid someone else)
- Yes, had sold sexual services (someone else paid you)
- Had before, but not in the past year

1. Have you been involved in group sexual activity (three or more people)?

- Yes, with all women
- Yes, with both men and women
- Never had

1. What information do you think are necessary regarding sexual health?

- Knowledge of condom use
- Knowledge of sexually transmitted infections (STI)
- Guidance on sexual pleasure
- Nothing needed
- Others, please indicate_____________________________________

***Sexual health status***

1. Have you experienced the following symptoms in the last year?

- Voiding dysfunction (frequent urination/urinary urgency/dysuria)
- Abnormal urethra secretions
- Vaginal inflammation
- Genital skin damage
- None of the above
- Others, please indicate_____________________________________

1. Have you ever been infected with the following diseases?

- Urethritis
- Vaginitis
- Cervicitis
- Pelvic inflammation
- Gonorrhea (gonorrhea urethritis)
- Genital warts
- Syphilis
- Genital herpes
- Hepatitis A
- Hepatitis B
- Hepatitis C
- None of the above
- Others, please indicate_____________________________________

1. If you experience the above-mentioned symptoms, what would you do?

- Go to a public hospital and seek help from health professionals (e.g., tertiary hospital)
- Go to a community hospital or community service centers
- Go to a private clinic for some medicine treatment
- Go to the pharmacy for advice
- Self-treatment
- Others, please indicate_____________________________________

1. Have you ever done the following tests? (Multiple options)

- Had STI testing before, please indicate details _____________________________
- Had gynecological examination before, details ____________________________
- Had HIV testing before, details ___________________________________
- Had other testing/screenings, details _________________________________
- None of the above

1. Reason for testing/ screenings:
   1. Why did you do the testing? (Multiple options)
   - Routine physical examination
   - Hope to better understand my health
   - Because I have many sexual partners
   - Because I do not feel well about myself
   - Because did not perform safer sex behaviors when having sex
   - Because not clear the health status of my partner
   1. How about the reason you did not do any testing?
   - Because I have no sexual activities
   - Because I have only one stable partner
   - Because the number of my sexual partners is very small
   - Because I feel I am good and healthy
   - Because I always perform safer sex behaviors
   - Because I do not know where to do testing
2. Where do you go for treatment or testing if you have sexual health concerns?

- STI specialists in tertiary hospitals
- STI specialists in community hospitals
- Private STI clinic
- Private gynecological clinic
- Beauty salon or health club
- Others, please indicate_____________________________________

1. In the last year, have you been looking for a doctor for sex counseling?

- Yes, please indicate_____________________________________
- No

1. What do you know about HPV (human papillomavirus)?

- Know nothing
- Know some
- Know most
- Fully know

1. What is your HPV vaccination status?

- Not vaccinated and not intended to be vaccinated
- Not vaccinated, but will do
- Already vaccinated with 2-valent HPV vaccine or vaccination in progress
- Already vaccinated with 4-valent HPV vaccine or vaccination in progress
- Already vaccinated with 9-valent HPV vaccine or vaccination in progress
- Others, please indicate_____________________________________

1. Who would you be willing to talk to about sexual health?

- With parents/siblings
- With intimate partner(s)
- With friends outside the community (e.g., LGBTQ community outsiders/peers)
- With friends inside the community (e.g., LGBTQ community insiders/peers)
- With friends met online
- With psychologist
- With health manager
- With healthcare staff
- Unwilling to talk sex or rarely talk sex
- Others, please indicate_____________________________________

1. Have you received any guidance on women’s sexual health?

- Never been exposed to relevant content
- Had some fixed sources, please indicate ______________________________
- Had some random sources, please indicate __________________________________
- Did not know where to get such information
- Others, please indicate_____________________________________

1. What are the main sources of information you have about sexual health?

- Books/magazines/television/radio
- Friends and/or colleagues
- Website information, please indicate_____________________________________
- WeChat official account/ Weibo, please indicate______________________________
- Healthcare staff at the hospital
- Community brochures
- Never seen such information
- Others, please indicate_____________________________________

1. Do you think the sexual health knowledge currently available have satisfied your needs?

- Cannot meet my needs at all
- Not quite able to meet needs
- Meet general needs
- Almost enough to meet my needs
- Fully meet my needs

1. What kind of sexual related guidance or other assistance do you think you need?

- Female sexual health knowledge
- Female guide to sexual pleasure
- Intimate relationship instruction
- Safer sex guidance
- Others, please indicate_____________________________________
- Nothing needed

**Positive Sexuality Scale**

*Choose the extent to which you agree or disagree with each of the following statements in the light of your actual partner relationship and sexual life experience.*

|  | **Strongly disagree** | | | | | **Strongly agree** | |
| --- | --- | --- | --- | --- | --- | --- | --- |
| 1. Sex brings a sense of fulfilment in my couple relationship | 1 | 2 | 3 | 4 | 5 | 6 | 7 |
| 1. Sex with my partner is a beautiful experience. | 1 | 2 | 3 | 4 | 5 | 6 | 7 |
| 1. Our intimate relationship is sexually stimulating | 1 | 2 | 3 | 4 | 5 | 6 | 7 |
| 1. Sex brings fun and joy in my couple relationship | 1 | 2 | 3 | 4 | 5 | 6 | 7 |
| 1. Sex with my partner is an exciting experience | 1 | 2 | 3 | 4 | 5 | 6 | 7 |

**New Sexual Satisfaction Scale-Short form**

*Thinking about your sex life during the last six months please rather your satisfaction with the follow aspects.*

|  | Not at all Satisfied | A Little Satisfied | Moderately Satisfied | Very Satisfied | Extremely Satisfied |
| --- | --- | --- | --- | --- | --- |
| 1. The quality of my orgasms. | 1 | 2 | 3 | 4 | 5 |
| 1. My “letting go” and surrender to sexual pleasure during sex. | 1 | 2 | 3 | 4 | 5 |
| 1. The way I sexually react to my partner. | 1 | 2 | 3 | 4 | 5 |
| 1. My body’s sexual functioning. | 1 | 2 | 3 | 4 | 5 |
| 1. My mood after sexual activity. | 1 | 2 | 3 | 4 | 5 |
| 1. The pleasure I provide to my partner. | 1 | 2 | 3 | 4 | 5 |
| 1. The balance between what I give and receive in sex. | 1 | 2 | 3 | 4 | 5 |
| 1. My partner’s emotional opening up during sex. | 1 | 2 | 3 | 4 | 5 |
| 1. My partner’s ability to orgasm. | 1 | 2 | 3 | 4 | 5 |
| 1. My partner’s sexual creativity. | 1 | 2 | 3 | 4 | 5 |
| 1. The variety of my sexual activities. | 1 | 2 | 3 | 4 | 5 |
| 1. The frequency of my sexual activity. | 1 | 2 | 3 | 4 | 5 |

**Female Sexual Function Index**

**INSTRUCTIONS**: These questions ask about your sexual feelings and responses during the past 4 weeks. Please answer the following questions as honestly and clearly as possible. Your responses will be kept completely conﬁdential. In answering these questions the following deﬁnitions apply:

**Sexual activity** -can include caressing, foreplay, masturbation and vaginal penetration.

**Vaginal penetration** is deﬁned as penetration (entry) of the vagina with any object (ﬁngers, sex toys,)

**Sexual stimulation** includes situations like foreplay with a partner, self-stimulation (masturbation), or sexual fantasy.

1. Over the past 4 weeks, how **often** did you feel sexual desire or interest?

- Almost always or always
- Most times (more than half the time)
- Sometimes (about half the time)
- A few times (less than half the time)
- Almost never or never

1. Over the past 4 weeks, how would you rate your **level** (degree) of sexual desire or interest?

- Very high
- High
- Moderate
- Low
- Very low or not at all

1. Over the past 4 weeks, how **often** did you feel sexually aroused ("turned on") during sexual activity or intercourse?

- No sexual activity
- Almost always or always
- Most times (more than half the time)
- Sometimes (about half the time)
- A few times (less than half the time)
- Almost never or never

1. Over the past 4 weeks, how would you rate your **level** of sexual arousal ("turn on") during sexual activity or intercourse?

- No sexual activity
- Almost always or always
- Most times (more than half the time)
- Sometimes (about half the time)
- A few times (less than half the time)
- Almost never or never

1. Over the past 4 weeks, how **confident** were you about becoming sexually aroused during sexual activity or intercourse?

- No sexual activity
- Very high confidence
- High confidence
- Moderate confidence
- Lower confidence
- Very low or no confidence

1. Over the past 4 weeks, how **often** have you been satisfied with your arousal (excitement) during sexual activity or intercourse?

- No sexual activity
- Almost always or always
- Most times (more than half the time)
- Sometimes (about half the time)
- A few times (less than half the time)
- Almost never or never

1. Over the past 4 weeks, how **often** did you become lubricated ("wet") during sexual activity or intercourse?

- No sexual activity
- Almost always or always
- Most times (more than half the time)
- Sometimes (about half the time)
- A few times (less than half the time)
- Almost never or never

1. Over the past 4 weeks, how **difficult** was it to become lubricated ("wet") during sexual activity or intercourse?

- No sexual activity
- Extremely difficult or impossible
- Very difficult
- Difficult
- Slightly difficult
- Not difficult

1. Over the past 4 weeks, how often did you **maintain** your lubrication ("wetness") until completion of sexual activity or intercourse?

- No sexual activity
- Almost always or always
- Most times (more than half the time)
- Sometimes (about half the time)
- A few times (less than half the time)
- Almost never or never

1. Over the past 4 weeks, how **difficult** was it to maintain your lubrication ("wetness") until completion of sexual activity or intercourse?

- No sexual activity
- Extremely difficult or impossible
- Very difficult
- Difficult
- Slightly difficult
- Not difficult

1. Over the past 4 weeks, when you had sexual stimulation or intercourse, how **often** did you reach orgasm (climax)?

- No sexual activity
- Almost always or always
- Most times (more than half the time)
- Sometimes (about half the time)
- A few times (less than half the time)
- Almost never or never

1. Over the past 4 weeks, when you had sexual stimulation or intercourse, how **difficult** was it for you to reach orgasm (climax)?

- No sexual activity
- Extremely difficult or impossible
- Very difficult
- Difficult
- Slightly difficult
- Not difficult

1. Over the past 4 weeks, how **satisfied** were you with your ability to reach orgasm (climax) during sexual activity or intercourse?

- No sexual activity
- Very satisfied
- Moderately satisfied
- About equally satisfied and dissatisfied
- Moderately dissatisfied
- Very dissatisfied

1. Over the past 4 weeks, how **satisfied** have you been with the amount of emotional closeness during sexual activity between you and your partner?

- No sexual activity
- Very satisfied
- Moderately satisfied
- About equally satisfied and dissatisfied
- Moderately dissatisfied
- Very dissatisfied

1. Over the past 4 weeks, how **satisfied** have you been with your sexual relationship with your partner?

- Very satisfied
- Moderately satisfied
- About equally satisfied and dissatisfied
- Moderately dissatisfied
- Very dissatisfied

1. Over the past 4 weeks, how **satisfied** have you been with your overall sexual life?

- Very satisfied
- Moderately satisfied
- About equally satisfied and dissatisfied
- Moderately dissatisfied
- Very dissatisfied

1. Over the past 4 weeks, how **often** did you experience discomfort or pain during vaginal penetration?

- Did not attempt vaginal penetration
- Almost always or always
- Most times (more than half the time)
- Sometimes (about half the time)
- A few times (less than half the time)
- Almost never or never

1. Over the past 4 weeks, how **often** did you experience discomfort or pain following vaginal penetration?

- Did not attempt vaginal penetration
- Almost always or always
- Most times (more than half the time)
- Sometimes (about half the time)
- A few times (less than half the time)
- Almost never or never

1. Over the past 4 weeks, how would you rate your **level** (degree) of discomfort or pain during or following vaginal penetration?

- Did not attempt vaginal penetration
- Very high
- High
- Moderate
- Low
- Very low or none at all

**The next phase of the investigation is intended**

If we are going to conduct a qualitative study about sexual health in the future, are you interested to join?

- Yes, contact information_____________________________________
- No

**「多元性身份女性的健康情况调查」**

# 第一部份–知情同意

**您被邀请参加的研究是由香港大学研究团队进行。在您决定是否参加此项研究前，您必需了解此项研究的目的及其有关资料。请仔细阅读下面的资料，如果您发现任何不明白的地方，请留言提出问题。**

1. 此次研究的目的是什么？

**此次研究目的是评估女性的健康情况和健康需求，其中不仅包含性与性别少数女性，也包含性多数(异性恋)女性。**

2. 如果我参加此项研究，我将要做什么？

**您须完成一份关于健康情况和健康需求调查的问卷，需时大约30分钟。随后，如果您有意愿的话，您会被邀请参加一对一访谈，大约30～60分钟。**

3. 如果我参加此项研究，会有什么风险？

**参加这项研究活动并不会为您带来任何已知的危险也不会令您感到不安。**

4. 我是否必须参与此项研究？

**您的参与全属自愿性质，如果您决定不参与或日后退出此项研究，将不会影响您的任何权利。如果您决定参加，您也可以不给予任何原因随时退出而不受任何惩罚。**

5. 我的个人资料会否受到保密？

**在研究期间所得的资料将会绝对保密，更不会给予研究组以外的人传阅。能识别您个人身份的资料将以您的参与者编号取代。**

6. 如欲知更多详情，有什么途径？

**香港大学研究操守委员会(HREC)已审查并通过此项研究，编号为：EA210325。**

**如果您想进一步了解此项研究的详情，欢迎您联络研究员：吴女士 (微信/邮箱……)。**

**若您对参与研究的权利有任何问题，请联络香港大学研究操守委员会。**

9. 若我同意参加此项研究，我该怎么做？

**若您同意参加此项研究，请确认以下事项：[多选题] ***

- 我确定已被告知有关此项研究的资料。
- 我明白此次参与属自愿性质，可随时退出而不需提出任何理由。
- 我明白我所提供的资料有可能被此项研究的负责团队查看；同时我确定本人的个人识别资料在所有书写文件及对外发表的文件上将受到绝对保密。
- 我同意参与上述研究。

# 第二部份-问卷问题

## 社会人口学资料

请您按照自己的实际情况填写，并在最符合自己情况的选项中打“√”进行选择或填空。

1. 年龄：_______周岁
2. 您的文化程度是？

- 小学及以下
- 初中
- 高中/中专
- 大专/本科
- 研究生及以上

1. 您的民族是？

- 汉族
- 壮族
- 回族
- 满族
- 藏族
- 其他，请说明_____________________________________

1. 您现在的职业是什么？（您在大部分的时间里所从事的工作？）

- 无业或待业
- 离休或退休
- 学生（全日制）
- 国家机关/群众团体/社会组织/企事业单位负责人
- 专业技术人员（科研/教职/医护/艺术家等）
- 办事人员和有关人员（行政/警察/消防等）
- 社会生产服务和生活服务人员（餐饮/销售/银行/地产等）
- 农民/牧民/渔民/林业生产及辅助人员
- 生产制造及有关人员（工人）
- 军人
- 其他，请说明_____________________________________

1. 您每月个人经济收入大约是？

- 无收入或少于1000元
- 1001~3000元
- 3001~5000元
- 5001~7000元
- 7001~9000元
- 9001~11000元
- >11000元

## 性身份认同

*自我认同或许会随时间发生变化，请就当下您的身份认同和既往的生活经历回答下列问题。所有答案无关对错，且所有回答都会保密，请如实填写。*

### 性别认同

1. 您出生指派性别（生理性别）是？

- 男性
- 女性
- 间性
- 其他，请说明_____________________________________

1. 您的身份证（通行证）上的性别是？

- 男性
- 女性

1. 您的性别认同是？

- 顺性别女性
- 顺性别男性（结束问卷）
- 跨性别女性
- 跨性别男性（结束问卷）
- 性别酷儿
- 流动性别
- 不能确定
- 其他，请说明_____________________________________

1. 您是在多大年龄完成性别自我认同的？（即明确知道自己性别）_____________岁
2. 从知道自己的性别认同以来，您的心理感受情况？（可多选）

- 自然发展，一切顺利
- 我的性别认同让我时常感到焦虑
- 我的性别认同让我时常感到抑郁
- 我的性别认同让我时常感到孤独
- 我有过自杀的想法，但无自杀行为
- 我不仅有过自杀的想法，也尝试过自杀行为，请说明______________________

### 性取向认同

1. 在情感（精神）方面吸引您的人是：

- 完全是女性
- 多数是女性
- 男女两性差不多
- 多数是男性
- 完全是男性
- 说不清

1. 在身体（性）方面吸引您的人是：

- 完全是女性
- 多数是女性
- 男女两性差不多
- 多数是男性
- 完全是男性
- 说不清

1. 您认为您的性取向是？

- 同性恋（只对同性产生爱情和性欲）
- 双性恋（只对异性产生爱情和性欲）
- 异性恋（对男女两性皆会产生爱情和性欲）
- 泛性恋（对任何性别皆能产生爱情和性欲）
- 不能确定
- 其他，请说明_____________________________________

1. 您是在多大年龄第一次明确自己的性取向？

_____________岁

1. 从知道自己的性取向以来，您的心理感受情况？（可多选）

- 自然发展，一切顺利
- 我的性取向让我时常感到焦虑
- 我的性取向让我时常感到抑郁
- 我的性取向让我时常感到孤独
- 我有过自杀的想法，但无自杀行为
- 我不仅有过自杀的想法，也尝试过自杀行为，请说明______________________

若非顺性别异性恋女性，则继续回答下列问题：

1. 您的出柜情况是？

- 未出柜，家人朋友都不知道
- 半出柜，仅面向朋友出柜
- 半出柜，仅面向家人出柜
- 半出柜，大部分人都知道，仍有同事/家人不知道
- 全面出柜，家人朋友都知道
- 其他，请说明_____________________________________

1. 在女女伴侣关系中，您认为自己属于什么（性）角色？

- T（tomboy，近似男性角色）
- P（po，婆，近似女性角色）
- H（half，一半一半）
- 无固定角色
- 不想被标签
- 其他，请说明_____________________________________

1. 您觉得您最能接受哪个称谓？

- 拉拉
- 蕾丝边（lesbian音译）
- 同性恋
- 同志
- 女女性行为者（women who have sex with women，WSW）
- 女双性恋
- 女泛性恋
- 其他，请说明_____________________________________

1. 您有无通过网络寻找性伴侣（约炮）？

- 有，请问网络途径是_____________________________________
- 无

## 亲密关系

1. 请选择一个最恰当的能够描述您当前亲密关系状态的选项：

- 未婚，单身
- 未婚，有伴侣（女性）
- 未婚，有伴侣（男性）
- 未婚，有伴侣（跨性别）
- 已婚（异性婚姻）
- 已婚（同性婚姻），请具体说明 _________________ *
- 离异
- 丧偶
- 其他，请说明_________________ *

1. 您与您伴侣之间的关系是属于？

- 彼此专一（彼此都只有对方一个性伴侣）
- 我专一，对方不专一
- 对方专一，我不专一
- 都不专一（彼此可以有不止一个伴侣，或开放关系）
- 我专一，但不清楚对方情况
- 我不专一，同时不清楚对方情况
- 其他，请说明_________________ *

1. 您自己对婚姻的看法是？（可多选）

- 可能会找异性结婚
- 可能会找Gay形婚
- 坚定等待同性婚姻合法
- 肯定不会进入异性婚姻
- 可能会选择意定监护
- 暂无想法
- 其他，请说明_____________________________________

## 性行为与性健康

*以下问题主要是了解您的性行为和性健康情况，建议您在一个安全私密的环境下填写。请根据您的实际情况选择最合适您自己的答案。*

### 性经历

1. 您有无自慰过？（能产生性欲唤起的自己对自己的刺激行为）

- 无（跳转至27）
- 有

1. 您第一次自慰是在多大年龄?

_______________岁

1. 在过去一年里，您自慰的频率是？

- 每天几次
- 每天1次
- 每周1~3次
- 每月1~3次
- 每2~3月1次
- 半年1次或更少

1. 您使用性用具自慰的情况？

- 从未使用过
- 有使用过

1. 您过往发生性行为的情况是？

- 从未跟任何人发生任何形式的性行为（跳过NSSS-S和PSS问卷）
- 有过性接触或其他性活动，但未发生过插入/纳入式性行为（口/阴道/肛门等）
- 有发生过插入式性行为（通过手/性用具/阴茎等）
- 其他，请说明_________________

1. 您发生第一次性行为是在多大年龄？

_______________岁

1. 您第一次性行为的对象是？

- 女性
  - 除了女生外，请问您有无同其ta性别的伙伴发生过性行为？
    - 无
    - 有，男性
    - 有，跨性别，请说明___________________________
- 男性
  - 除了男生外，请问您有无同其ta性别的伙伴发生过性行为？
    - 无
    - 有，女性
    - 有，跨性别，请说明___________________________
- 其ta性别，请说明_____________________________________
  - 除此之外，请问您有无和其ta性别的人发生过性行为?
    - 无
    - 有，女性
    - 有，男性
    - 有，跨性别，请说明___________________________

1. 您最喜欢的性行为方式是哪一种？（可多选）

- 前戏（接吻/抚触）
- 口交（口爱）
- 手指交
- 拳头交
- 自慰（自我探索）
- 阴道交
- 肛门交
- 性器具/性玩具
- 以上都喜欢
- 其他，请说明_____________________________________

1. 目前您是否有固定性伴侣（与自己互有感情和稳定性关系的伴侣）？

- 有，男性
- 有，女性
- 有性伴侣，但不是固定性伴侣
- 没有性伴侣

1. 迄今为止，您曾与多少个不同的男性发生过性行为？_____________人
   - 最近六个月，您与男性发生过性行为？（手交/口交/阴道交/肛交等）
     - 无
     - 有，请问人数是_____________
2. 迄今为止，您曾与多少个不同的女性发生过性行为？_____________人
   - 最近六个月，您与女性发生过性行为？（手交/口交/阴道交/肛交等）
     - 无
     - 有，请问人数是_____________

### 性安全行为

1. 您一般使用何种方式进行安全防护？（可多选）

- 使用安全套/避孕套/手指套/口交套等（选择后方可填写下一题）
- 自己清洁手/口/阴道/肛门/性用具等
- 双方都清洁手/口/阴道/肛门/性用具等
- 未特意采用过任何安全防护措施
- 其他，请说明_____________________________________

1. 您经常使用的安全套类型是？（可多选）

- 从未使用过，原因是_________________*
- 常规安全套/避孕套
- 女用安全套
- 手指套
- 口交套
- 其他，请说明 _________________*

1. 您一般从什么途径获得安全套/手指套/口交套？（可多选）

- 线下超市/便利店
- 线下药店/性保健品店/自动售货机
- 免费领取的
- 线上购买
- 都是伴侣准备
- 其他，请说明_____________________________________

1. 最近六个月，您发生插入式性行为时使用安全套/指套/口交套的频率如何？

- 每次都用安全套/指套/口交套
- 经常使用安全套/指套/口交套（超过一半的时间）
- 偶尔使用安全套/指套/口交套（少于一半的时间）
- 从来不用安全套/指套/口交套

1. 您最近一次插入式性行为时使用安全套了吗?

- 有使用安全套
- 有使用手指套
- 有使用口交套
- 使用了其他类型安全套，请说明 _________________ *
- 未使用

1. 若没有每次都使用安全套/指套，则原因是？（可多选）

- 当下没有可用安全套
- 我不愿意用
- 伴侣不愿意用
- 认为没有危险
- 不能接受套套的气味
- 价格太贵
- 觉得影响体验（缺乏快感）
- 其他，请说明_____________________________________

1. 您有无性交易的经历？

- 从未有过
- 有，“买”过性服务（您付钱给别人）
- 有，“卖”过性服务（别人付钱给您）
- 近一年没有过

1. 您有无参与过多人性活动（3人及以上的群体性行为活动）？

- 有，全是女性
- 有，有男有女
- 从未有过

1. 关于性安全，您认为哪些方面的信息需要了解？

- 安全套等的正确使用
- 性传播疾病的知识
- 性爱技巧
- 我不需要
- 其他，请说明_____________________________________

### 性健康状况

1. 最近一年，您是否出现过下列相关症状？

- 排尿异常（尿频/尿急/尿痛）
- 尿道分泌物异常
- 阴道炎症
- 生殖器皮肤黏膜破损、溃疡或增生
- 以上均无
- 其他，请说明_____________________________________

1. 您是否曾经感染过下列疾病？

- 尿道炎
- 阴道炎
- 宫颈炎
- 盆腔炎
- 淋病（淋菌性尿道炎）
- 尖锐湿疣
- 梅毒
- 生殖器疱疹
- 甲型肝炎
- 乙型肝炎
- 丙型肝炎
- 其他，请说明_____________________________________
- 以上均无

1. 如果您出现上述相关症状，您会选择？

- 去大医院就诊（如：三甲医院）
- 去社区医院就诊（或：社区服务中心）
- 去私立诊所求医
- 去药店咨询
- 自我治疗
- 其他，请说明_____________________________________

1. 您曾经是否做过下列检测？（可多选）

- 做过性病检测，请说明_____________________________________
- 做过妇科检查，请说明_____________________________________
- 做过HIV检测，请说明_____________________________________
- 做过其它检测，请说明_____________________________________
- 以上均无

1. 检测原因：
   - 您去做检测的原因是？（可多选）
   - 常规例行体检
   - 希望更好的了解自己的健康状况
   - 因为性伴侣数量比较多
   - 因为自我感觉身体不舒服
   - 因为没有使用安全措施
   - 因为不清楚伴侣的健康情况
   - 您没有做过任何检测的原因是？
   - 因为没有性生活
   - 因为只有一个固定性伴
   - 因为性伴数量很少
   - 因为自我感觉很健康
   - 因为一直坚持使用安全措施，如安全套/指套
   - 因为不知道去哪里检测
2. 您一般去做检测或治疗，选择的机构场所是在？

- 公立医院的性病专科
- 公立医院的妇科
- 私立性病诊所
- 私立妇科病诊所
- 美容院或养生会所
- 其他，请说明_____________________________________

1. 最近一年，您是否去寻找过医生进行性咨询？

- 有，原因是_____________________________________
- 无

1. 您对HPV（人乳头瘤病毒）了解多少？

- 完全不了解
- 了解一些
- 大部分了解
- 完全了解

1. 您的HPV疫苗接种情况？

- 未接种，且不打算接种
- 未接种，但有计划接种
- 已接种2价HPV疫苗或接种进行中
- 已接种4价HPV疫苗或接种进行中
- 已接种9价HPV疫苗或接种进行中
- 其他，请说明_____________________________________

1. 您最愿意与谁交流“性”健康相关问题？

- 父母或亲友
- 伴侣
- 社群内（圈内）朋友
- 社群外（圈外）朋友
- 网友（线上聊天）
- 心理咨询师
- 健康管理师
- 医务工作者
- 不喜欢谈论或很少谈论
- 其他，请说明_____________________________________

1. 您有无接受过有关女性性健康的指导资讯？

- 从未接触到相关内容
- 有固定渠道获取相关内容，请说明_____________________________________
- 其他随机渠道获取相关内容，请说明____________________________________
- 不了解
- 其他，请说明_____________________________________

1. 您获得性健康相关知识的信息来源主要是？

- 书本/杂志/电视/广播
- 朋友和（或）同事
- 网站网页信息，请说明_____________________________________
- 微信公众号/视频号/微博，请说明_____________________________________
- 医院的医护人员
- 社区宣传册
- 从未看到相关信息
- 其他，请说明_____________________________________

1. 您认为目前能获取的性健康知识是否能满足您的需求？

- 完全不满足
- 不太满足
- 不确定
- 比较满足
- 完全满足

1. 您认为您需要哪些性相关的知识指导或其他帮助？

- 女性性健康
- 女性性愉悦
- 性爱安全指导
- 亲密关系指导
- 其他，请说明_____________________________________
- 都不需要

## 【PSS】-性积极量表

请结合您的实际伴侣关系及性生活经历，选择您同意或不同意以下每个表述的程度

|  | **非常不同意** | |  |  |  | **非常同意** | |
| --- | --- | --- | --- | --- | --- | --- | --- |
| 1. 性给我的亲密关系带来满足感。 | 1 | 2 | 3 | 4 | 5 | 6 | 7 |
| 1. 与伴侣发生性关系是一种美好的体验。 | 1 | 2 | 3 | 4 | 5 | 6 | 7 |
| 1. 我们的亲密关系有助于性刺激。 | 1 | 2 | 3 | 4 | 5 | 6 | 7 |
| 1. 性给我的亲密关系带来乐趣和愉悦感。 | 1 | 2 | 3 | 4 | 5 | 6 | 7 |
| 1. 与伴侣发生性关系是一种令人兴奋的经历。 | 1 | 2 | 3 | 4 | 5 | 6 | 7 |

## 【NSSS-S】性满意度量表

（当题目**您有无自慰过？**选择**[有]**时，**或者**当题目**您过往发生性行为的情况是？**选择**[有过性接触或其他性活动，但未发生过插入式性行为（插入或被插入口/阴道/肛门等）]、[有发生过插入式性行为（通过手/性用具/阴茎等）]、[其他，请说明]**中的其中一个选项时，**此题才显示**）

想想您最近6个月的性生活，对下面描述各项的满意度进行评定。

|  | **一点也不满意** | **有一点满意** | **一般满意** | **非常满意** | **极其满意** |
| --- | --- | --- | --- | --- | --- |
| 1. 我性高潮的质量。 | 1 | 2 | 3 | 4 | 5 |
| 1. 性行为中我能“释放自我”并享受性愉悦。 | 1 | 2 | 3 | 4 | 5 |
| 1. 我回应我伴侣的性表达方式。 | 1 | 2 | 3 | 4 | 5 |
| 1. 我身体的性功能。 | 1 | 2 | 3 | 4 | 5 |
| 1. 性行为后我的心情 | 1 | 2 | 3 | 4 | 5 |
| 1. 我提供给我伴侣的性愉悦。 | 1 | 2 | 3 | 4 | 5 |
| 1. 性行为中我付出的与我得到的之间的平衡。 | 1 | 2 | 3 | 4 | 5 |
| 1. 性行为中我伴侣情绪放得开。 | 1 | 2 | 3 | 4 | 5 |
| 1. 我伴侣达到性高潮的能力。 | 1 | 2 | 3 | 4 | 5 |
| 1. 我伴侣性方面的新奇性。 | 1 | 2 | 3 | 4 | 5 |
| 1. 我性行为的多样性。 | 1 | 2 | 3 | 4 | 5 |
| 1. 我性行为的频率。 | 1 | 2 | 3 | 4 | 5 |

## 【FSFI-女性性功能】

（当题目**您有无自慰过？**选择**[有]**时，**或者**当题目**您过往发生性行为的情况是？**选择**[有过性接触或其他性活动，但未发生过插入式性行为（插入或被插入 口/阴道/肛门等）]、[有发生过插入式性行为（通过手/性用具/阴茎等）]、[其他，请说明]**中的其中一个选项时，**此题才显示**）

以下问题是询问您最近4周来的性感受和性反应，请您尽可能诚实、清楚地回答下列问题（每个题目只能选一项），对您的回答我们会完全保密。回答这些问题时需要用到以下定义：

- 性活动：可包括亲吻、性爱抚、自慰和阴道性交。
- 性交：指阴茎或手指或性器具插入（进入）阴道。
- 性刺激：包括与性伴侣的性前戏，自我刺激（自慰），或性幻想。
- 性欲或性兴趣：是一种想要性经历的感受，愿意接受伴侣发起的性活动，渴望或幻想做爱。
- 性唤起：是一种包括生理和精神两方面的性兴奋，也可包括生殖器发热或兴奋，润滑（湿度增加）或肌肉收缩。

1. 在过去的4周中，您感受到有性欲（性欲望）的**频率**是？

- 几乎总是
- 大部分时间（一半以上的时间）
- 有时（大概一半的时间）
- 较少的时间（少于一半的时间）
- 几乎没有或没有

1. 您如何评价您过去4周的性欲**程度**（强度）？

- 非常高
- 高
- 中等
- 低
- 很低或没有

1. 在过去的4周中，您在性活动或性交时感受到被唤起（性兴奋）的**频率**是？

- 无性活动
- 总是或几乎总是
- 大部分时间（一半以上的时间）
- 有时（大概一半的时间）
- 较少的时间（少于一半的时间）
- 几乎没有或没有

1. 在您过去的4周的性活动或性交时，您如何评价自己**性兴奋的程度**？

- 无性活动
- 非常高
- 高
- 中等
- 低
- 很低或没有

1. 在过去的4周中，您对自己能在性活动或性交中被性唤起的**信心**有多大？

- 无性活动
- 非常高的信心
- 高的信心
- 中等度的信心
- 较低的信心
- 很低或没有信心

1. 在您过去的4周的性活动或性交中，您对自己的性唤起（兴奋）感到满意的**频率**是？

- 无性活动
- 总是或几乎总是
- 大部分时间（一半以上的时间）
- 有时（大概一半的时间）
- 较少的时间（少于一半的时间）
- 几乎没有或没有

1. 在您过去的4周的性活动或性交中，您的阴道变得润滑（湿润）的**频率**是？

- 无性活动
- 总是或几乎总是
- 大部分时间（一半以上的时间）
- 有时（大概一半的时间）
- 较少的时间（少于一半的时间）
- 几乎没有或没有

1. 在您过去的4周的性活动或性交中，您的阴道变得润滑（湿润）有多**困难**？

- 无性活动
- 极度困难或不能变湿
- 非常困难
- 困难
- 轻度困难
- 无困难

1. 在您过去的4周的性活动或性交中，您能维持阴道润滑（湿润）直到性活动或性交结束的**频率**是？

- 无性活动
- 总是或几乎总是
- 大部分时间（一半以上的时间）
- 有时（大概一半的时间）
- 较少的时间（少于一半的时间）
- 几乎没有或没有

1. 在您过去的4周的性活动或性交中，您维持阴道润滑（湿润）直到性活动或性交结束的**困难程度**是？

- 无性活动
- 极度困难或不能变湿
- 非常困难
- 困难
- 轻度困难
- 无困难

1. 在过去的4周中，当您有性刺激或性交时，您达到性高潮的**频率**是？

- 无性活动
- 总是或几乎总是
- 大部分时间（一半以上的时间）
- 有时（大概一半的时间）
- 较少的时间（少于一半的时间）
- 几乎没有或没有

1. 在过去的4周中，当您有性刺激或性交时，要达到高潮的**困难程度**是？

- 无性活动
- 极度困难或不能
- 非常困难
- 困难
- 轻度困难
- 无困难

1. 在过去的4周中，您对您在性活动或性交中达到高潮的**满意度**是？

- 无性活动
- 非常满意
- 中度满意
- 满意和不满意各占一半
- 中度不满意
- 非常不满意

1. 在过去的4周中，您对您和您的性伴侣在**性活动过程中的情感上的亲密度**的满意度是？

- 无性活动（任何形式均无）
- 非常满意
- 中度满意
- 满意和不满意各占一半
- 中度不满意
- 非常不满意
- 无性伴侣（仅有自慰等性活动）

1. 在过去的4周中，您对您和您伴侣的**性关系的满意度**是？

- 非常满意
- 中度满意
- 满意和不满意各占一半
- 中度不满意
- 非常不满意
- 无性伴侣

1. 在过去的4周中，您对性生活的**整体满意度**是？

- 非常满意
- 中度满意
- 满意和不满意各占一半
- 中度不满意
- 非常不满意

1. 在过去的4周中，当阴道被插入**期间**，您感到不适或疼痛的**频率**是？

- 没有尝试性交
- 总是或几乎总是
- 大部分时间（一半以上的时间）
- 有时（大概一半的时间）
- 较少的时间（少于一半的时间）
- 几乎没有或没有

1. 在过去的4周中，当插入式性行为**结束后**，您感到阴道不适或疼痛的**频率**是？

- 没有尝试性交
- 总是或几乎总是
- 大部分时间（一半以上的时间）
- 有时（大概一半的时间）
- 较少的时间（少于一半的时间）
- 几乎没有或没有

1. 在过去的4周中，您如何评价插入式性行为**期间及结束以后**的阴道不适或疼痛**程度**？

- 没有尝试性交
- 很高
- 高
- 中等
- 低
- 非常低或没有

# 写在最后

非常感谢您的参与！接下来我们会继续开展一对一的质性访谈，如果我们继续对“性健康”进行更深入的定性研究（访谈），请问您有兴趣加入吗？

- 是，联系方式_____________________________________
- 否
