# Supplementary material for: Sexual health and sexual behaviours in Chinese women of varied sexual identities: a sequential mixed methods study
Source: Sex Reprod Health Matters. 2026 Feb 9;33(1):2624200. doi: 10.1080/26410397.2026.2624200 (PMC13112868; doi:10.1080/26410397.2026.2624200)
Supplement: Supplemental tables S1 and S2 [file ZRHM_A_2624200_SM9342.docx]

**Supplemental Tables**

# Table S1 Correlation matrix of all variables assessed in the larger project

|  | **Variables** | 1 | 2 | 3 | 4 | 5 | 6 | 7 | 8 | 9 | 10 | 11 | 12 | 13 | 14 | 15 |
| --- | --- | --- | --- | --- | --- | --- | --- | --- | --- | --- | --- | --- | --- | --- | --- | --- |
| 1 | **NSSS-S** | 1 |  |  |  |  |  |  |  |  |  |  |  |  |  |  |
| 2 | **PSS** | 0.537^**^ | 1 |  |  |  |  |  |  |  |  |  |  |  |  |  |
| 3 | **FSFI** | 0.686^**^ | 0.634^**^ | 1 |  |  |  |  |  |  |  |  |  |  |  |  |
| 4 | ***Desire*** | 0.278^**^ | 0.233^**^ | 0.514^**^ | 1 |  |  |  |  |  |  |  |  |  |  |  |
| 5 | ***Arousal*** | 0.570^**^ | 0.475^**^ | 0.815^**^ | 0.534^**^ | 1 |  |  |  |  |  |  |  |  |  |  |
| 6 | ***Lubrication*** | 0.412^**^ | 0.233^**^ | 0.697^**^ | 0.235^**^ | 0.532^**^ | 1 |  |  |  |  |  |  |  |  |  |
| 7 | ***Orgasm*** | 0.477^**^ | 0.288^**^ | 0.704^**^ | 0.151^*^ | 0.447^**^ | 0.315^**^ | 1 |  |  |  |  |  |  |  |  |
| 8 | ***Satisfaction*** | 0.629^**^ | 0.373^**^ | 0.760^**^ | 0.189^**^ | 0.499^**^ | 0.386^**^ | 0.603^**^ | 1 |  |  |  |  |  |  |  |
| 9 | ***Pain*** | 0.314^**^ | 0.442^**^ | 0.593^**^ | 0.158^*^ | 0.309^**^ | 0.459^**^ | 0.268^**^ | 0.306^**^ | 1 |  |  |  |  |  |  |
| 10 | **GAD-7** | -0.206^**^ | -0.164^**^ | -0.314^**^ | -0.04 | -0.157^**^ | -0.129^*^ | -0.257^**^ | -0.320^**^ | -0.190^**^ | 1 |  |  |  |  |  |
| 11 | **PHQ-9** | -0.261^**^ | -0.169^**^ | -0.351^**^ | -0.019 | -0.181^**^ | -0.177^**^ | -0.221^**^ | -0.404^**^ | -0.259^**^ | 0.801^**^ | 1 |  |  |  |  |
| 12 | **RSES** | 0.236^**^ | 0.250^**^ | 0.362^**^ | 0.034 | 0.239^**^ | 0.149^*^ | 0.240^**^ | 0.337^**^ | 0.143^*^ | -0.507^**^ | -0.578^**^ | 1 |  |  |  |
| 13 | **PSQI** | -0.220^**^ | -0.187^**^ | -0.324^**^ | -0.009 | -0.163^**^ | -0.101 | -0.255^**^ | -0.335^**^ | -0.287^**^ | 0.445^**^ | 0.564^**^ | -0.377^**^ | 1 |  |  |
| 14 | **SSRS** | 0.402^**^ | 0.230^**^ | 0.261^**^ | 0.037 | 0.130^*^ | 0.155^**^ | 0.172^**^ | 0.338^**^ | 0.234^**^ | -0.250^**^ | -0.376^**^ | 0.376^**^ | -0.283^**^ | 1 |  |
| 15 | **QOL** | 0.450^**^ | 0.293^**^ | 0.510^**^ | 0.068 | 0.315^**^ | 0.289^**^ | 0.316^**^ | 0.495^**^ | 0.374^**^ | -0.580^**^ | -0.694^**^ | 0.721^**^ | -0.540^**^ | 0.541^**^ | 1 |

Note: ^*^ *p*<0.05; ^**^ *p*<0.01. Correlations were performed using Pearson correlations. Results with significant estimates are highlighted in red font.

NSSS-S (New Sexual Satisfaction Scale-Short form); PSS (Positive sexuality scale); FSFI (Female Sexual Function Index); GAD-7 (7-item Generalized Anxiety Disorder scale); PHQ-9 (9-item Patient Health Questionnaire); RSES (Rosenberg Self-Esteem Scale); PSQI (Pittsburgh Sleep Quality Index); SSRS (Social Support Rating Scale); QOL (WHOQOL-BREF: World Health Organization Quality of Life–abbreviated short version

# Table S2 Overview of birthplace, current residence, and residence change (*N*=509)

| Province of residence | Place of birth | Current residence |
| --- | --- | --- |
| Economically developed area ^a^ |  |  |
| Beijing | 11(2.2) | 43(8.4) |
| Shanghai | 11(2.2) | 49(9.6) |
| Fujian | 13(2.6) | 4(0.8) |
| Jiangsu | 23(4.5) | 32(6.3) |
| Tianjin | 6(1.2) | 9(1.8) |
| Zhejiang | 15(2.9) | 15(2.9) |
| Hong Kong | 4(0.8) | 31(6.1) |
| Macau | 0 | 3(0.6) |
| Taiwan | 2(0.4) | 0 |
| *Total* | *85(16.7)* | *186(36.5)* |
| Economically moderate area ^b^ |  |  |
| Guangdong | 85(16.7) | 144(28.3) |
| Chongqing | 12(2.4) | 10(2.0) |
| Hubei | 30(5.9) | 28(5.5) |
| Shandong | 21(4.1) | 9(1.8) |
| Inner Mongolia | 5(1.0) | 1(0.2) |
| Shaanxi | 8(1.6) | 3(0.6) |
| Anhui | 16(3.1) | 4(0.8) |
| Hunan | 27(5.3) | 14(2.8) |
| Liaoning | 17(3.3) | 10(2.0) |
| Sichuan | 27(5.3) | 20(3.9) |
| Jiangxi | 26(5.1) | 8(1.6) |
| Henan | 56(11.0) | 34(6.7) |
| Hainan | 1(0.2) | 3(0.6) |
| Ningxia | 1(0.2) | 1(0.2) |
| Xinjiang | 4(0.8) | 0 |
| Yunnan | 6(1.2) | 2(0.4) |
| Qinghai | 1(0.2) | 1(0.2) |
| Jilin | 8(1.6) | 0 |
| Shanxi | 17(3.3) | 8(1.6) |
| *Total* | *368(72.3)* | *300(58.9)* |
| Economically underdeveloped area ^c^ |  |  |
| Hebei | 16(3.1) | 9(1.8) |
| Guizhou | 10(2.0) | 4(0.8) |
| Guangxi | 14(2.8) | 5(1.0) |
| Heilongjiang | 11(2.2) | 4(0.8) |
| Gansu | 5(1.0) | 1(0.2) |
| *Total* | *56(11.0)* | *23(4.5)* |
| Residence movement |  |  |
| Upward movers ^d^ | 194(38.1) | |
| *Moderate to Developed* | *142(27.9)* | |
| *Underdeveloped to Developed* | *18(3.5)* | |
| *Underdeveloped to Moderate* | *34(6.7)* | |
| Lateral movers ^e^ | 241(47.3) | |
| *Maintain in Developed* | *26(5.1)* | |
| *Maintain in Moderate* | *211(41.5)* | |
| *Maintain in Underdeveloped* | *4(0.8)* | |
| Downward movers ^f^ | 74(14.5) | |
| *Developed to Moderate* | *55(10.8)* | |
| *Developed to Underdeveloped* | *4(0.8)* | |
| *Moderate to Underdeveloped* | *15(2.9)* | |

^a^: According to the gross domestic product (GDP) per capita, “Economically developed areas” refer to provinces where per capita GDP exceeds CNY 100,000, Chinese Yuan (CNY).

^b^: “Economically moderate areas” refer to the provinces where per capita GDP between CNY 50,000 and CNY 100,000).

^c^: “Economically underdeveloped areas” refer to the provinces where per capita GDP less than CNYU50,000.

^d^: “Upward movers” refer to individuals who moved to more economically developed areas.

^e^: “Lateral movers” refer to individuals who moved between areas with similar economic levels.

^f^: “Downward movers” refer to individuals who moved to less economically developed areas.
